# Multiple cell types guided by neurocytes orchestrate horn bud initiation in dairy goats

**DOI:** 10.1186/s12711-025-00981-3

**Published:** 2025-07-01

**Authors:** Hegang Li, Mengmeng Du, Xiaokun Lin, Xinxin Cao, Lu Leng, F. M. Perez Campo, Dongliang Xu, Lele Hou, Xiaoxiao Gao, Jianyu Zhou, Ming Cheng, Jianguang Wang, Qinan Zhao, Yin Chen, Feng Yang, Jinshan Zhao

**Affiliations:** 1 Qingdao Agricultural University, Qingdao, China; 2 University of Cantabria, Torrelavega, Santandery, Spain; 3 Qingdao Institute of Animal Husbandry and Veterinary Medicine, Qingdao, China; 4 Inner Mongolia Shengjian Biotechnology Co., Ltd, Hohhot, China; 5 Inner Mongolia Academy of Agricultural & Animal Husbandry Sciences, Hohhot, China; 6 China Meat Food Comprehensive Research Center, Beijing, China

## Abstract

**Background:**

Horn development is a key ruminant trait involving multi-cell type coordination via molecular pathways. This study used scRNA-seq to analyze cellular heterogeneity and fate trajectories during early horn bud niche formation, revealing key gene expression profiles. Combining with hematoxylin–eosin (HE) staining and immunohistochemical analysis, we further verified the asynchronous developmental pathways of key cells in the skin tissue of fetal goat horn bud at induction (embryonic day (E) 50; E50), organogenesis (E60), and cytodifferentiation (E70) stages, and demonstrated the signal transmission routes for the development of early horn buds.

**Results:**

We revealed temporal and spatial differences of the main signal transmission of horn bud development combining with existing literatures. We speculated that multiple cell types under the guidance of nerve cells collaborated on horn bud initiation in dairy goats. In detail, neural cells receive initial horn bud signals, stimulating hair follicle cell degeneration and transmitting to dermal cells, which evolve through intermediates, amplify signals to epithelial cells, and differentiate into mesenchymal cells. Nerve cell branches also trigger neural crest cell production/migration, working with chondrocytes to promote keratinocyte differentiation for horn bud formation. In addition, we further identified the early horn bud developmental specific events, including the screening of biological functions, signaling pathways and key candidate genes.

**Conclusions:**

This study employed scRNA-seq to characterize cell fate trajectories and gene expression profiles in goat fetal horn buds. Histological comparisons between hornless and horned fetuses revealed cellular heterogeneity in epithelial, dermal, nerve, and hair follicle cells, with pseudo-time analysis identifying distinct differentiation paths. Dermal and epithelial cell transcriptional dynamics were critical for horn bud initiation (branch 1), supported by immunohistochemistry. Keratinocyte and nerve cell state transitions actively regulated horn development, with asynchronous cell development visualized via immunohistochemistry. Functional enrichment analyses (GO/KEGG) highlighted neural crest development and keratinocyte differentiation pathways, identifying candidate genes (*EGR1*, *ZEB2*, *SFRP2*, *KRT10*, *FMOD*, *CENPW*, *LDB1*, *TWIST1*) involved in horn morphogenesis. These findings advance understanding of goat horn development and genetic determinants.

**Supplementary Information:**

The online version contains supplementary material available at 10.1186/s12711-025-00981-3.

## Background

Goat horns are skin derivatives in the morphological structure which are hollow paired apparatus located on the frontal bone with a skeletal core covered by the integument. Able to be used as a natural weapon, horns play a crucial role in the defense against predators as well as in sexual selection by their use in intra-male competition in the field [1–3]. However, horns have no necessary and useful function in domesticated ruminants [4]. Ruminants with horns pose a danger when interacting with humans and flock mates [5]. They are also more likely to destroy farm facilities, reduce meat quality, or cause serious injuries to the udder [6], resulting in significant losses for the goat farming industry. Therefore, the study of horn formation and the presence or absence of horn is critical not only for the study of natural and sexual selection but also for the breeding of hornless goat breeds to promote modern goat farming.

The embryonic period is crucial for the differentiation and formation of horns. While horn development has been well-characterized in Merino sheep [7], the cellular and molecular mechanisms governing caprine horn formation during embryogenesis remain poorly understood. E70 goat fetuses exhibit distinct horn bud differentiation [2, 8], though no studies explicitly report the exact timing of horn bud formation for the goat. Previous studies have demonstrated that the presence of ruminant horn may have a common genetic basis, namely from neural crest stem cells [2]. In addition, horn specialization may be closely associated with the epithelial–mesenchymal transition promoted by the differentiation of neural crest stem cells [9]. While the *TWIST1*, *SOX10*, *NGFR*, and *HOXD* gene clusters were closely related to neural crest stem cell migration. And in particular, the downstream *HOXD* gene clusters can act as a major regulator of neural crest cell migration which over-regulated the specific elements of horn development in the cranial region [10]. Wagner et al. further confirm that *SOX10* and *NGFR* are expressed positively in the embryonic horn bud of sheep by immunohistochemical analysis [9]. *SNAI1* and *TFAP2A* are also specifically highly expressed in the fetal horn bud, but not in the adjacent skin tissue [9]. In addition, the genes *FFAR4*, *PRSS22*, and *SCUBE2* have been also validated which associated with the epithelial–mesenchymal transition [11–13]. It can be suggested that these genes can regulate the differentiation of neural crest cells, and also have a certain effect on the migration and differentiation of epithelial-mesenchymal cells. However, the precise molecular mechanisms governing epithelial-mesenchymal-neural crest cell interactions during embryogenesis remain inadequately characterized.

*FOXL2* is found to be a gene involved in horn development and involves in negative regulation of horn bud differentiation in goats [2, 3]. *PISRT1* is a testicular inhibitory factor that can inhibit the expression of *SOX9* [14]*.* Meanwhile, the interaction between *SOX9* and *FOXL2* might further regulate horn development and formation [15]. However, whether these genes have a regulatory effect on embryonic goat bud development needs to be further verified.

Single-cell transcriptome sequencing technology (scRNA-seq) is an unbiased method that can sequence thousands of cells in a single experiment, as well as obtain thousands of individual features per cell and provide ultrahigh-resolution transcriptomes of animal tissues and organs [16]. Especially, scRNA seq has been used to analyze and compare testicular tissues in different periods and species including goats [17–19]. Furthermore, several groups have also successfully used this technology to reveal the molecular machinery underlying cashmere goat hair follicle development [20–22]. To sum up, these recent discoveries further emphasize the prospective application of this technology in goat horn bud development-related research. The changes in cell state and the molecular regulatory mechanism of gene expression changes during the early development of the hornless to horned state of dairy goat embryos are still unclear. Therefore, it is necessary to explore the regulatory mechanism of horn bud development at the single-cell transcriptional level. In our study, scRNA-seq analysis was performed on the horn buds and corresponding forehead skin of Laoshan dairy goat fetuses to clarify the gene expression profile of goat horn buds to inquire whether the candidate genes for horn identified in previous studies affecting horn bud development, and also to discover new and more important cell types and candidate genes. This would allow us to address the paucity of information regarding the cellular heterogeneity and the molecular pathways underlying key cell fate decisions during horn bud formation.

## Methods

### Experimental animals

The four pregnant ewes of Laoshan dairy goats in this study were from the Sijichun dairy goat farm in Jimo City (Qingdao, China) and were reared using the dairy goat breeding standard. Two, two and four goat fetuses respectively at embryonic day 50 (abbreviated as E50), E60, and E70 stages were delivered by cesarean section after the gestating goats were anesthetized with thiazide hydrochloride. The genotypes for the horn of these 8 fetuses was detected by PCR [23] to ensure that both genotypes were evenly distributed. The forehead skin of hornless fetuses and horn bud skin of horned fetuses were collected at those three different periods, and each skin tissue block (5 mm × 5 mm × 1 mm) was excised into two parts. In detail, one part of E70 horned and hornless skin tissues was used for scRNA-seq, and the other part together with E50 and E60 skin tissues were used for immunofluorescence analysis. An overview of overall experimental design was displayed in Table 1. All experimental procedures designed for this study were approved by the Experimental Animal Management Committee of Qingdao Agricultural University.

**Table 1:** An overview of overall experimental design

Development stage	E50	E60	E70
Sample size (mm)	5 × 5 × 1	5 × 5 × 1	5 × 5 × 1
Detection methods	Hematoxylin–eosin staining(HE), Immunohistochemistry(IMH)	HE, IMH	HE, IMH, scRNA-seq
Sampe numbers for sc-RNA seq	0	0	2 samples each from horned and hornless embryos
Genotypes for embryos	1 embryo each for PIS^+/+^ and PIS^±^ genotypes	1 embryo each for PIS^+/+^ and PIS^±^ genotypes	2 embryos each for PIS^+/+^ and PIS^±^ genotypes

### Single-cell suspension preparation

Skin tissue blocks (5 mm × 5 mm × 1 mm) were excised from the forehead skin and horn bud skin of E70 fetuses, immediately transferred to ice-cold DMEM/F12 medium (Catalog No. C11330500BT, Gibco, Beijing, China) containing 50 U/ml penicillin and 50 mg/ml streptomycin (Catalog No. SV30010, Hy Clone, Logan, UT). After washing three times with DMEM/F12 to remove any blood cells, the skin tissue was disintegrated into individual cells ready for sequencing. The disintegration method was as follows. For E70 fetuses samples, skin tissue was first cut into 3 mm slices, and the obtained skin tissue was first incubated with 2 mg/ml collagenase IV for 20 min at 37 °C. Tissue was then mechanically separated into single-cell suspensions using 1 ml pipette tips, and the obtained single-cell suspensions were then filtered through a 40 μm nylon cell filter to remove hair debris. The cell viabilities of the samples were ensured to be greater than 85% to facilitate the further step of single cell library construction.

### Single-cell library construction and sequencing

Single cell suspensions of horn bud skin were obtained through the above steps and placed into the 10 × Genome single cell RNA sequencing platform (10 × Genome, CA, USA) for single cell barcoding and library preparation. All software and algorithms used in this study are listed (Table 2). Briefly, single-cell suspensions prepared above were counted using a hemocytometer (TC20, Bio-Rad, Hercules, CA, USA), and cell suspension adjusted to 1000 cells/μl before barcoding, obtaining approximately 7000 cells per stage. After single cell library construction, the Illumina Hi Seq X 10 sequencer barcode was used according to the manufacturer's instructions with 10 × barcoded gel beads, using the 10 × Genomic Chromium Single Cell 3 library and gel bead package v2 (10 × Genomics, Inc., Pleasanton, CA, USA, 120,237) and the 10 × Genomic Chromium barcode system was used to construct a 10 × barcoded cDNA library. Sequencing was performed using an Illumina Hi Seq X10 sequencer (Illumina, San Diego, CA, USA) to generate 150 bp (PE150) reads for downstream analysis.

**Table 2:** The software and algorithms used in this study

Name	Company	Information
CellRanger 2.2.0	10 × Genomics	https://support.10xgenomics.com/single-cell-gene-expression/software/overview/welcome
Seurat v3.2.0		https://satijalab.org/seurat/vignettes.html
Goat reference genome	Ensemble	https://asia.ensembl.org/Capra_hircus/Info/Index
Monocle 2.26.0		http://cole-trapnell-lab.github.io/monocle-release/ docs/
R 4.6.2	R Project	https://www.r-project.org/
Metascape		http://metascape.org/
KEGG		https://www.genome.jp/kegg/mapper/color.html
CellphoneDB		https://github.com/Teichlab/cellphonedb

### 10 × Genomics scRNA-seq data processing

Processing was performed using the standard Cell Ranger (v2.2.0) pipeline according to the official 10 × Genomics guidelines. The generated raw base call files were first converted to fastq files using the cell fast function. The goat ARS1 reference genome was downloaded to integrate as the reference genome. The cell manager count function was used to perform mapping, filtering of low-quality cells, barcode counting, and unique molecular identifier (UMI) counting. After using the standard Cell Ranger pipeline, according to the official user guide. The generated gene expression matrix files were analyzed using the Seurat (V3.2.0) software package using filtered cell functions for quality control against detection of genes. After normalization and data scaling, different datasets were integrated using the Run Multi CCA function. Dimensionality reduction analysis was performed using UMAP [24] and different clusters of cells were identified using the Find Clusters function. These clusters consisting of specifically expressed genes were refreshed using the find marker function and the parameter “min.pct = 0.25. Use = 0.25”, which is the smallest log2 multiple of the mean expression of a gene in a group relative to the mean expression of all other groups, with a default value of 0.25.

### Unsupervised clustering analysis

Quality control and Seurat software (v3.2.0) was used for single-cell RNA sequence according to the Cell Ranger pipeline, based on the online guide, and the R environment was using for cell clustering analysis. We used the “filtered gene bc matrices” file generated by Cell Ranger as the input file for Seurat. For each dataset, we first filtered the cells with less than 200 unique genes detected and those with less than 3 genes detected, and then we used the “filtered cells” function to remove cells with less than 1750 total genes (n Genes) detected. After normalization, the variable genes were calculated for each dataset and downstream clustering analysis was performed.

To compare transcriptome profiles at two different developmental time points, we merged two different datasets using the “Run Multi CCA” function implemented in Seurat. Run Multi CCA uses a typical correlation analysis to remove the variation caused by the sample source. After the datasets were aligned, we performed a clustering analysis on the integrated datasets based on the UMAP and t-distributed random neighborhood embedding algorithms implemented in Seurat. To identify genes specifically expressed in the clusters, we used the “discover all markers” function implemented in Seurat to calculate the clustering markers and annotated the cell clusters identified by UMAP based on previously reported typical marker gene expression [20–22].

To subcluster cell clusters of interest for in-depth analysis and/or downstream differentiation trajectory construction, we used the Seurat implemented “Subset Data” function to extract clusters of interest. The extracted subclusters were then re-run through the Seurat pipeline, which provides higher resolution for dissecting cellular heterogeneity among particular cell types.

### Single-cell pseudo-time lineage trajectory reconstruction

Single-cell gene reconstruction analysis was performed using the Monocle 2.26.0 [25, 26] package according to the online tutorial (http://cole-trapnell-lab.github.io/monocle-release/docs/). Single slice objects were constructed using the new Cell Data Setset object, and variable genes identified by Seurat were used as sorting genes to sort cells along the trajectory in pseudo-time. The DDRTree method was used for dimensionality reduction. To analyze gene expression differences between different cell branches, we used the BEAM function and identified differently expressed genes (DEGs) using q-values. A branch-specific gene expression heatmap was plotted with the plot genes branched heatmap function, and different gene sets were calculated according to k-means clustering. RNA velocity analysis was carried out following the official velocyto.R guide (https://github.com/velocyto-team/velocyto.R). In short, the velocyto.py pipeline was employed to extract unspliced and spliced reads from the bam files produced by CellRanger. Subsequently, velocyto.R was applied to compute the velocity vectors. Finally, the “show.velocity.on.embedding.cor” function was used to incorporate the RNA velocity vectors into the monocle object. CellphoneDB [27, 28] was used to analyze communication networks between different cell types, including ligands, receptors, and their interactions. Euclidean distances between all cell pairs within subgroups were calculated and presented as box plots to visualize their distributions.

### Enrichment analysis

To further understand the functions of DEGs between horn buds and forehead skin tissues in goats, Gene Ontology (GO) and the Kyoto Encyclopedia of Genes and Genomes (KEGG) were used for enrichment analysis. This was carried out on the Metascape [29] and KEGG Mapper [30, 31] tools. All analyses were deemed substantially enriched if the p-value was less than 0.05. All p values were adjusted using Benjamini and Hochberg’s FDR correction method.

### Immunofluorescence analysis

Skin tissue samples from horn buds or corresponding regions at E50, E60, and E70 were selected and placed in tissue fixative solution. The skin tissue was subsequently washed, dehydrated, and embedded in paraffin. The paraffin-embedded skin tissue sections were stained with hematoxylin–eosin (HE), and the remaining paraffin-embedded skin tissues were used for immunofluorescence analysis. For immunofluorescence analysis, the samples were firstly deparaffinized in xylene and further rehydrated in an ethanol solution. Antigen retrieval was performed in 0.01 M sodium citrate buffer at 96 °C. After a permeabilization procedure in 0.5 M Tris-HCI buffer supplemented with 0.5% Triton X-100 (Catalog No. T8200, Sorlabio, Beijing, China) for 10 min, the slides were then blocked with 3% BSA and 10% donkey serum (Catalog No. AR0009, Boster, Wuhan, China) in 0.5 M Tris-HCI buffer for 30 min. Primary antibodies diluted in the blocking buffer were incubated with the slides at 4 °C overnight, and then corresponding secondary antibodies were added and incubated at 37 °C for 1 h. 4′,6-diamidino-2-phenylindole (DAPI) was used to stain the nuclei. The pictures were taken using a Nikon AR1 confocal system (Nikon, Tokyo, Japan). The slides were finally mounted with neutral resins, and pictures were captured with an Olympus BX51 microscope imaging system (Olympus, Tokyo, Japan). All the primary and secondary antibodies used in this study are listed (Table 3).

**Table 3:** The primary antibodies and secondary antibodies used in this study

Name	Company	Information
Rabbit monoclonal anti-LEF1	Abcam	Cat# ab137872, RRID:NA
Rabbit monoclonal Anti-Lum	Abcam	Cat# ab168348, RRID:NA
Rabbit monoclonal antiCytokeratin 15	Abcam	Ca#ab52816,RRID:AB 869863
Rabbit polyclonal anti-SOX9	Millipore	Cat# AB5535, RRID:AB 2239761
Goat Anti-Rabbit IgG H&L (Alexa Fluor® 488)	Servicebio	Cat# GB25303, RRID:AB 2910224

## Results

### ScRNA-seq identified different cell types in developing dairy goat horn buds

**Fig. 1:**
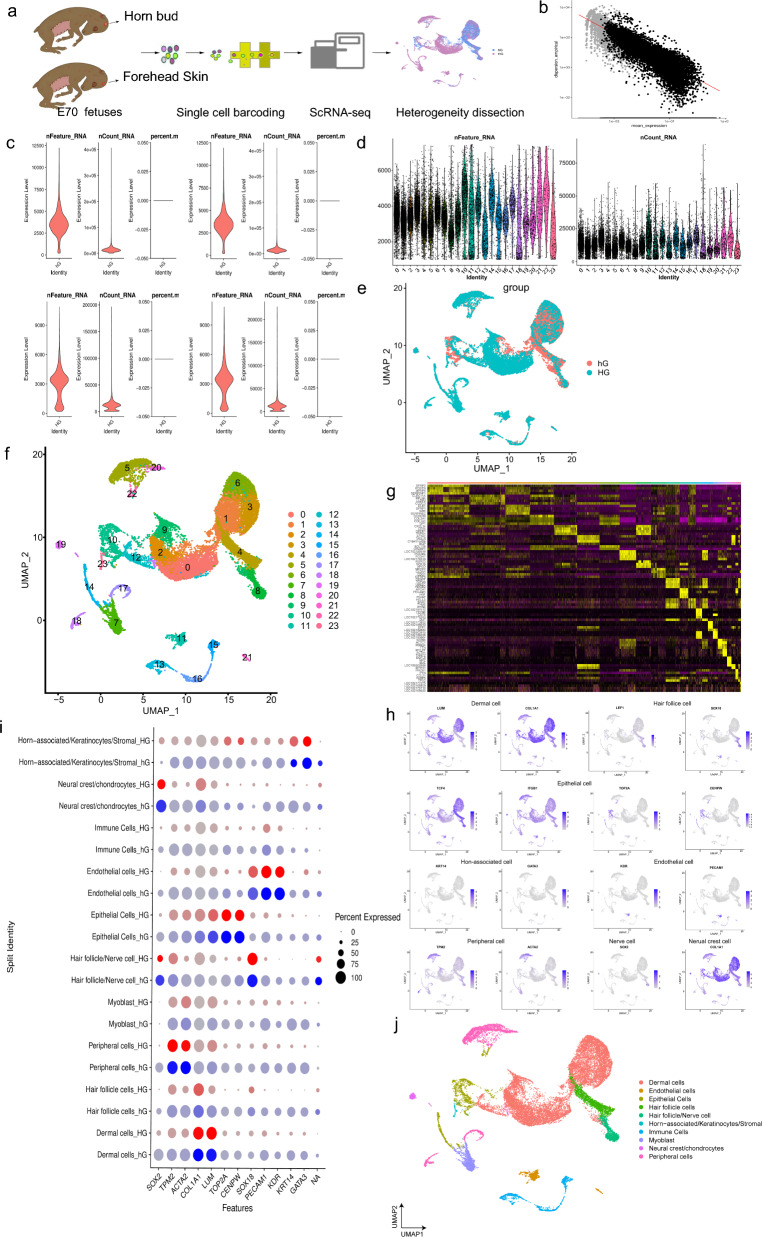
scRNA-seq delineated cellular heterogeneity during dairy goat horn bud development. **a** Overall experimental design. **b** Gene dispersion empirical after quality control of scRNA-seq. **c** The sample data after quality control, Hg1, Hg2, hg1, and hg2 respectively. **d** The overall situation after sample merging. **e**, **f** UMAP plot of all single cells labelled with developmental time. Cells from different developmental time points are color coded. **g** Heat map of gene expression. **h** Expression of marker genes in different cell types. **i** Dot plot of representative marker genes for different cell clusters. The color intensity represents log1p-transformed expression level (the darker the color, the higher the expression level), and the dot size represents the positive cell percentage (count > 0). **j** UMAP plot of all single cells label with cell groups according to their marker gene expression

To provide in-depth insights into the molecular profiles during dairy goat horn buds development and the core cell fate transitions, scRNA-seq was used to analyze the transcript expression profiles (Fig. 1a). We included the horn bud skin of horned fetuses and forehead skin of hornless fetuses from E70 in goats respectively, denoted by “Hg” and “hg”, and obtained ethical approval (see Additional file 1: Fig. S1a). Principal component analysis (PCA) and ANOVA of genes were then performed on four single-cell samples. The results showed that the 9864 genes of the two different traits were highly variable (see Additional file 1: Fig. S1b and Additional file 9: Table S1). We proceeded more reliable screening of all the samples and compared the capture of the number of molecules changes from hornless to horned. More than 289 million raw reads were captured, and overlapping information of 23 674 cell distribution and gene expression were retained for further analysis after strict quality control (see Additional file 1: Fig. S1c and Additional file 9: Table S2). Both the quality control results and the overall expression levels of the samples met the requirements for subsequent analysis (Fig. 1b–d).

In addition, to investigate the heterogeneity of cells during the development of horn buds, we performed UMAP clusters of all the single cells (Fig. 1e). The results showed that partial cells overlapped in the two different developmental stages, but the expression of cells in horned stage was more significant in the whole process and further indicated that horn bud might form during the E70 embryo developmental time. Based on the enrichment of marker gene expression previously reported in mice or other species, the major cell lineage of hornless to horned development was further divided into 23 distinct cell clusters (Fig. 1f; see Additional file 1: Fig. S1d). Collectively, these data establish the classification of different clusters and the selective gene expression pattern of dairy goats. There is the heat map of the top 10 specific marker genes in Fig. 1g, which showed analysis results of high-expressed marker genes. The different clusters were annotated by published datasets through the “single R” package, Cell Ranger database, and references, and re-clustering in major cell clusters. The expression levels of markers, including dermal cell lineage markers *LUM* and *COL1A1* [32, 33], the epithelial cell lineage markers *TCF4* [34–36], *ITGB1* [37–39], *TOP2A* [40, 41] and *CENPW* [42] (the latter two genes served as markers for the abnormal hyperplasia of the epithelial cells in the horn buds), endothelial cell expressing *KDR* and *PECAM1* [43], peripheral cell expressing *TPM2* and *ACTA2* [44], keratinocytes expressing *KAT14* and stromal *GATA3* [45–47], hair follicle expressing *LEF1* and *SOX18* [48, 49], immune cell expressing *RGS* and *FCER1G* [50, 51], neural crest cell expressing *COL1A1* [32, 49], and nerve cell expressing *SOX2* [52]*.* We identified a series of well-recognized marker genes that clustered revealing major cell groups (Fig. 1h; see Additional file 1: Fig. S1e). Briefly, clusters 1, 2, 3, 6, 9, and 12 expressed high levels of the dermal cell lineage markers *LUM* and *COL1A1.* Clusters 10, 14, and 22 expressed high levels of the epithelial cell lineage markers *TOP2A* and *CENPW.* Furthermore, several important cell clusters were also identified. These included endothelial cell clusters expressing *KDR* and *PECAM1* (clusters 11 and 21), peripheral cell clusters expressing *TPM2* and *ACTA2* (clusters 5, 17, 18, and 21), horn-associated/keratinocytes/stromal cell clusters expressing *KAT14* and *GATA3* (clusters 2, 3, 10, and 11), hair follicle clusters expressing *LEF1* and *SOX18* (clusters 4 and 8), immune cell clusters expressing *RGS* and *FCER1G* (clusters 13, 15, and 16), neural crest cell clusters expressing *COL1A1* (clusters 18 and 19) and nerve cell clusters expressing *SOX2* (clusters 8 and 9). We further screened all the marker genes to obtain more classical marker genes and gained the atlas about the expression of different cell types of marker genes (Fig. 1i). Interestingly, the same significantly expressed genes were expressed slightly differently in hornless and horn traits respectively, suggesting that changes in cell state affected gene levels differently during horn bud development. It was also worth noting that some clusters showed time-dependent accumulation, thus deciphering the process of cell differentiation at a particular time point.

To further verify the cellular heterogeneity revealed by UMAP analysis, we performed consensus clustering analysis on cells derived from the same lineage, re-clustering into the same module, thus further verifying key cell clusters (Fig. 1j). More importantly, the transcriptional characteristics of each cell type were delineated, and more cell type-specific marker genes were identified during the horn bud development of dairy goats. Furthermore, we explored some genes that were only expressed in certain cells, such as *COL1A1*, *KDR*, *LEF1*, *LUM*, *TOP2A*, and *ACTA2*. These significantly expressed genes, especially over-expressed in each functional cluster, may be functional candidate genes associated with horn bud development (see Additional file 1: Fig. S1f). In summary, we successfully identified the major cell types at single-cell resolution and characterized their cell type-specific gene expression pattern providing potential biological markers for future research.

### Recapitulation of development of dairy goat horn buds based on key cell trajectory changes and interactions

**Fig. 2:**
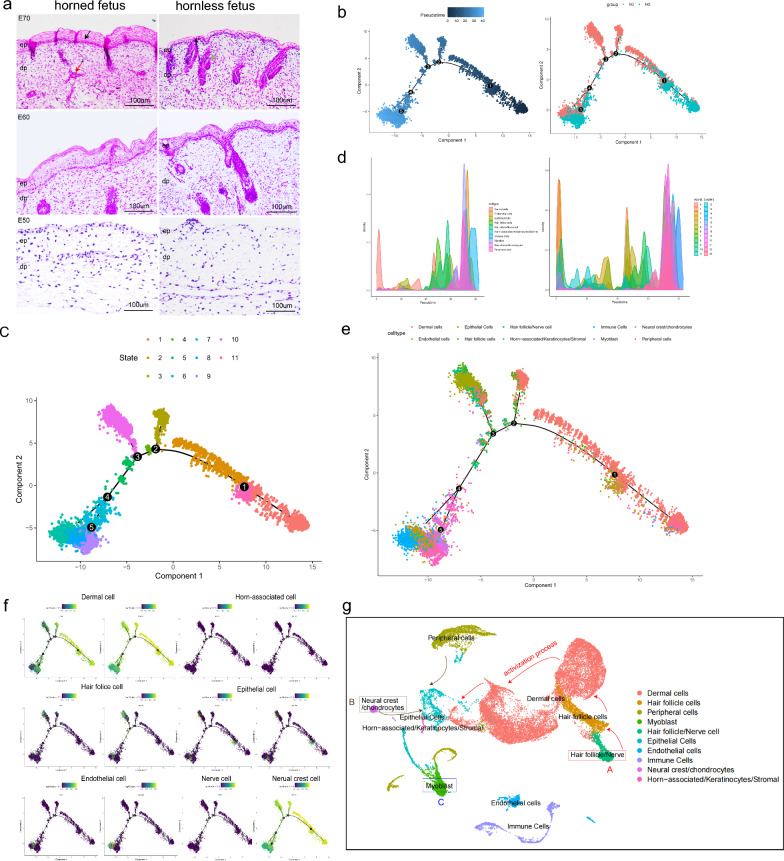
Cell lineage developmental trajectories delineated by pseudo-time trajectory inference analyses. **a** Horn buds with multiple epithelial layers for E70, E60, and E50 fetuses. **b** Developmental trajectory of different cell lineage along pseudo-time. Cells were color-coded with developmental time points (left panel) and groups identified by Monocle (right panel), respectively. **c** Based on cell expression density different periods and cell types. **d**, **e** Cell differentiation trajectories along pseudo-time. Cells were color-coded with cell types (left panel) and cell clusters (right panel) by Monocle, respectively. **f** Dynamic changes of major cell type marker genes. **g** Sequence map of cell differentiation and information transmission

After analyzing the characterization of major cell clusters, we investigated the effect of cell heterogeneity on horn bud development. By examining the well-defined anatomical structures of fetal horn bud tissue samples from various stages, the interaction and function of key cell lines in dairy goat horn bud were illustrated by combining with our UAMP-identified clusters. Macroscopically, we observed and compared histological changes in E50, E60, and E70 embryonic horn buds using HE staining to analyze their morphological characteristics (Fig. 2a). Comparing the phenotype of the samples revealed similar morphology between the fetal horn bud and forehead skin epithelium of E60 and E70 embryos. However, there was no significant change observed in skin tissue from E50 fetuses. Tissue slides further confirmed this conclusion. In detail, the number of epithelial cell layers in the E70 fetal horn bud region was significantly thicker than that in the forehead skin tissue. Especially, the epidermis in the region of the horn bud was up to 8 layers thick with keratinocyte vacuolation. In contrast, forehead skin had a thinner epidermis with only one layer of vacuolated keratinocytes. Moreover, the dermis in both the horn bud and forehead skin consisted mainly of immature collagen without any attached structures. However, during early gestation (E70), thick bundles of mesenchymal fibers were present below the horn bud middle to deep dermis. We speculated that these mesenchymal structures may have increased in size over time. Based on the previous reports, we speculated that these structures might be nerve bundles, observing that they were exclusively seen below the horn bud of a horn fetus but were not present in the forehead skin of the same fetus during the same period. In addition, the tissue below the forehead skin of the hornless fetus was clearly defined, with several hair follicles piled up, but there was no evidence below the horn bud. We also proved a similar pattern in the tissue structure of E50 and E60 fetal dairy goats. In summary, dermal cells, epithelial cells, keratinocytes, nerve cells, and hair follicles may be involved in the process of hornless to horned development.

After conducting a macroscopic comparison of the morphology of hornless and horn states, we investigated the intermediate states of key cells, changes in cell fate, and interactions among these key cells. In an unsupervised manner, pseudo-time analysis was employed to maximize transcriptional similarity to explore the dynamics of cell state transitions and the sequential occurrence of these states (Fig. 2b). The sub-population within cell classification was distributed throughout the entire tissue with significant overlap observed in each cluster, indicating their universality across various stages of horn bud development in dairy goats. For all cell lineages, the pseudo-time trajectory displayed five branch points and revealed 11 states of the horn bud development of the dairy goat (Fig. 2c). Additionally, we observed differential density changes in key cell types during horn bud development, suggesting that distinct cellular lineages may play indispensable roles throughout this process (Fig. 2d). Notably, when the cells were color-coded with their corresponding developmental stages, they also displayed an ordered pattern along pseudo-time (Fig. 2d). To further verify the trajectory inference analyses in the major cell lineages, we performed RNA velocity analyses since RNA velocity can be used to infer the developmental directionality by distinguishing the un-spliced and spliced mRNAs [53]. The results showed that the majority of the RNA velocity vectors displayed obvious directions toward the branch endpoint (see Additional file 2: Fig. S2), verifying our trajectory inference analyses.

Subsequently, by analyzing the cell fate trajectory of horn bud formation, 7 crucial cell lineages were observed playing a potential important role across five development time points along pseudo-time (see Additional file 3: Fig. S3a, b and Additional file 9: Table S3). Since all the cell clusters been successfully characterized, the dermal lineage cell clusters, the epithelial lineage cell clusters, the endothelial lineage cell clusters, the horn-associated/keratinocytes/stromal lineage cell clusters, the hair follicle/nerve lineage cell clusters, and the neural crest cells/cartilage lineage cell clusters were then selected to infer the cell lineage development trajectory (Fig. 2e; see Additional file 3: Fig. S3c). We determined the dynamic changes of these cell types contained in each key fate branch during the whole process of horn bud development (Fig. 2f; see Additional file 3: Fig. S3d). In detail, nerve cells acted as the first signal of horn bud development that stimulated gradually hair follicle and dermal cells to migrate and differentiate in branch1 (states 1, 2, and 11). Meanwhile, these cells transmitted developmental signals to other key cells in the early stage of horn bud development. Next, hair follicle cells further developed and proliferated, and after dermal cells experienced a variety of complex intermediate forms, epithelial cells began to differentiate, proliferation, and migration in branch 2 (states 2, 3, and 4). Furthermore, nerve cells stimulated the migration of neural crest cells, which made epithelial cells further migrate to mesenchymal cells gradually in branch 3 (states 4, 5, and 10). Subsequently, the continuous migration and differentiation of mesenchymal cells might play a key regulatory role in horn bud development in branch 4 (states 5, 6, and 7). At the same time, neural crest cell differentiation was a brief and important period that stimulated the proliferation of chondrocytes in the frontal skin. Under the combined action of various processes such as neural crest cell migration stimulation, epithelial cell migration and differentiation to mesenchymal cells, and chondrocyte development, keratinocyte cell development may further promote the changes of bone morphology and skin keratinocyte in branch5 (states 7, 8, and 9) (Fig. 2g). In summary, the development of early embryonic horn buds was inseparable from the change of cell state and differentiation.

### Delineating the transcription characteristics and development pathways during the epithelial and dermal cell fate decisions

**Fig. 3:**
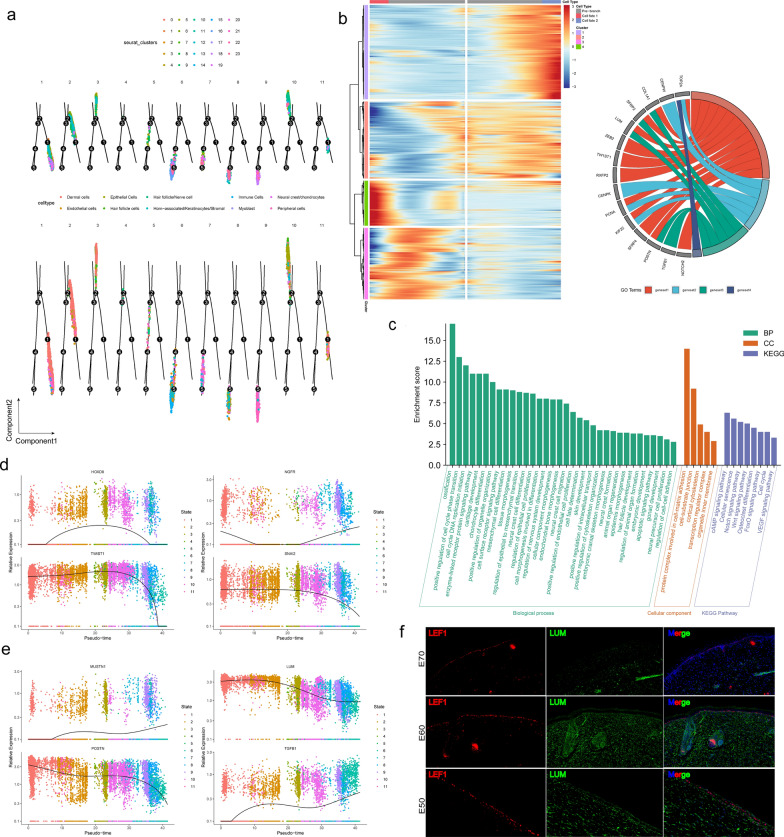
Pseudo-time trajectory analysis delineated molecular profiles during fate commitment. **a** Main branches of horn bud development. **b** Pseudo-time expression heatmap during branch1 commitment. The four gene sets were determined by k-means clustering according to their expression patterns. The right circular plot is used to display the relationships between gene data. The regions in different colors in the plot represent different gene sets (geneset). The gene names are labeled on the outer circle, and the differently—colored bands inside show the association relationships between gene sets and genes. **c** GO biological processes or KEGG pathway description in branch1. **d**, **e** Visualization of dairy goat orthologs of horn bud characteristic genes at different stages along pseudo-time. **f** immunofluorescence analysis of LUM/LEF1 expression in the embryonic horn bud

After revealing the different cell fate decisions in the horn bud, we decided to focus on the key branch points of the developmental trajectory. Different branch points during horn bud development were presented based on pseudo-time trajectory inference analysis. We concluded that cell expression changes during the transition from a hornless to a horned state. Based on DEGs dynamic analysis along pseudo-time, our results indicated that significantly different genes might affect the state of cell expression (Fig. 3a). Therefore, we chose branch 1 as the key point for our main analysis. In branch 1, we could detect that the development state of dermal cells and epithelial cells played a vital role, and the cell changes presented three different development directions. We analyzed all the differential gene expression in branch 1 and mapped the heat map of the gene expression (Fig. 3b; see Additional file 9: Table S4). The results showed that there were 4 different gene sets in branch 1, and each gene set showed 3 different developmental stages, namely pre-branch, cell fate 1, and cell fate 2. In addition, there were significant differences in the expression levels of DEGs at different periods, including that highly expressed genes in gene set 1 were mainly distributed in cell fate 2, highly expressed genes in gene set 2 were mainly distributed in pre-branch and cell fate 2, and highly expressed genes in gene set 3 and gene set 4 were mainly distributed in cell fate 1. As expected, dermal cell and epidermal cell lineages may be closely associated with initiating signals for horn formation as the starting point for development from hornless to horned. Therefore, characterizing the transcriptional characteristics and developmental pathways of epithelial and dermal cells were critical to the changes in intracellular expression levels.

Subsequently, GO enrichment analysis and KEGG analysis showed that “epithelial to intermediate, mesenchymal cell transition”, “keratinocyte differentiation”, and “regulation of epithelial cell proliferation” were significantly enriched (Fig. 3c; see Additional file 9: Table S5), while comparisons of GO terms revealed that each gene-set was different in biological function (see Additional file 4: Fig. S4a, b). To gain in-depth insight into the molecular profiles during epithelial and dermal cell fate commitment, characteristic gene expression patterns along the pseudo-time were further analyzed (see Additional file 4: Fig. S4c, d). For gene sets 2 and 3, we found transiently elevated expression levels of *SNAI2*, *NGFR*, *TWIST1*, and *HOXD8*, and enrichment of the GO terms of “regulation epithelial to mesenchymal transition” and “mesenchymal cell differentiation” for gene set 3 and “response to fibroblast growth factor”, “skin development” and “morphogenesis of an epithelium” for gene-set 2 (Fig. 3d). For gene sets 1 and 4, a series of regulation cell function genes were enriched, including *MUSTN1* and *TGFB1* were gradually increased in dermal and epithelial cells (Fig. 3e). However, *LUM* and *POSTN* expression level were gradually decreased along pseudo time (Fig. 3e). Therefore, these genes may play important regulatory roles during dermal and epithelial cell fate decisions.

*LUM* is an important extracellular matrix component and a member of the small molecule leucine proteoglycan family [54]. This gene plays a role in regulating collagen fiber synthesis and epithelial cell migration. *LEF1* works as a transcription factor to regulate Wnt signaling pathway and may play a role in hair follicle differentiation and morphogenesis [55]. As a marker gene of dermal cells, *LUM* is stably expressed during most transitional periods of horn bud development. *LEF1* acts as the marker gene of epidermal cells, for which obvious dynamic changes in the expression pattern were shown (see Additional file 4: Fig. S4e). The results showed that important changes occurred in the developmental pathways of the two different cell lineages during goat horn bud formation. Meanwhile, immunofluorescence analysis also confirmed this conclusion (Fig. 3f; see Additional file 4: Fig. S4f). In this study, we identified the expression of *LUM* and *LEF1* in the skin tissue of horn buds by immunofluorescence labeling. *LUM* was indeed highly expressed in the skin extracellular matrix, which proved that it was involved in the proliferation and differentiation of skin cells. *LEF1* was stably expressed in the nuclei of cylindrical cells in the basal layer of skin epidermis and follicle papilla cells. These results would indicate that *LEF1* was involved in the proliferation and differentiation of epidermal cells and the morphogenesis of hair follicles as a transcription factor, which was consistent with the situation in the hair follicles of mice and cashmere goats [56, 57]. However, its relationship with the formation and development of horn buds would need further investigation. Overall, immunofluorescence analysis validated the results of our single-cell transcriptome analysis, which enabled us to comprehensively study detailed developmental pathways involved in horn bud morphogenesis, and cell fate locus analysis provided valuable insights into the molecular pathways of horn bud cell development.

### Revealing the developmental pathways during keratinocyte and nerve cells fate commitment

**Fig. 4:**
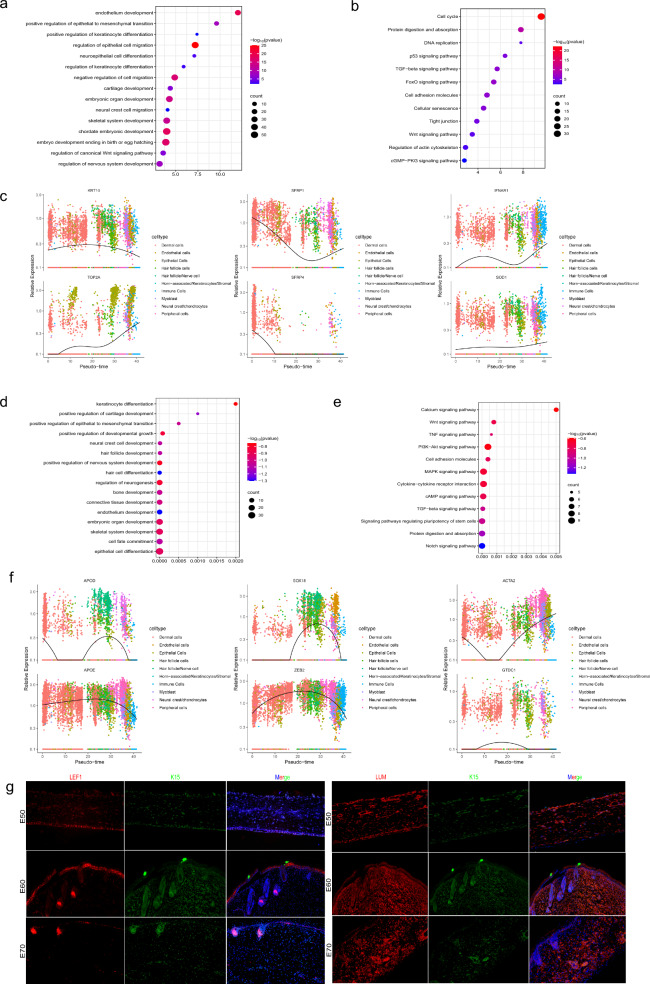
Analysis of gene function enrichment in keratinocyte and nerve cells fate commitment. **a** The main function of GO enrichment differential genes of keratinocyte. **b** KEGG analysis of keratinocyte. **c** Visualization of the DEGs in keratinocyte. **d** The main function of GO enrichment differential genes of nerve cells. **e** KEGG analysis of nerve cells. **f** Visualization of the DEGs in nerve cells. **g** Immunofluorescence analysis of LEF1/K15 and LUM/K15 expression in the horn bud

After describing the cell fate determination of dermal and epidermal cell lineages, other key cell populations were explored. Based on the description of cell heterogeneity and pseudo-time locus analysis, we further revealed the cell fate commitment of keratinocytes and nerve cells. To investigate the important functions of keratinocytes and nerve cells during horn development, we performed GO and KEGG analysis of their respective cluster marker genes. GO analysis revealed significant enrichment of keratinocyte lineages (clusters 2, 3, 10, and 11) in biological process categories (see Additional file 9: Table S6 and Additional file 5: Fig. S5a), including positive regulation of epithelial to mesenchymal transition, cartilage development, regulation of canonical Wnt signaling pathway, regulation of keratinocyte differentiation, and neural crest cell migration; in the cellular component category, they were enriched in the basal part of the cells, tight junction, extracellular matrix, and chromosome, centromeric region; in the molecular function category, they were enriched in cell adhesion molecule binding and growth factor binding (Fig. 4a). KEGG analysis of the keratinocyte marker genes indicated significant enrichment in the cell cycle, PI3K-Akt signaling pathway, TGF-β signaling pathway, p53 signaling pathway, and Wnt signaling pathway (Fig. 4b). These functions may further promote the differentiation of keratinocytes and horn-associated cells, and release factors related to horn formation to stimulate the migration and differentiation of other cells. When analyzing the DEGs along the pseudo-time trajectory, we found *TOP2A* and *KRT10* were up-regulated in keratinocytes, and yet in dermal and epithelial cell differentiation, *SFRP1* showed a pattern of first decreasing and then increasing, *SFRP4* exhibited a linear decline (Fig. 4c). *SOD1* expression slowly increased, and *IFNAR1* displayed cyclical fluctuations. Further research is needed to confirm whether these genes were involved in the formation and development of horns.

At the same time, to further investigate the important role of nerve cells in horn bud development, we annotated the use of GO for different clusters of nerve cells and performed KEGG function analysis (see Additional file 9: Table S7 and Additional file 5: Fig. S5b). As expected, GO analysis enriched with connective tissue development, hair follicle development, skeletal system development, and keratinocyte differentiation for horn bud growth, indicating these biological processes may relate to horn bud formation and development (Fig. 4d). At the same time, the KEGG pathways of these genes mainly enriched to the Wnt, TGF β, Notch, MAPK, and PI3K-Akt signaling pathways (Fig. 4e). These results suggested that nerve cells were involved in these biological processes and promoted the formation and development of horn buds. Notably, *APOD*, *SOX18*, *ZEB2*, and *APOE* were up-regulated in the development of hair follicle cells, nerve cells and keratinocytes. While the expression levels of *GTDC1* and *ACTA2* have significant stages of change during horn bud development (Fig. 4f). It suggested that these genes regulated the development of horn bud in the early stage.

Interestingly, we found that there were functional overlaps as well as functional differences in the highly expressed genes and enrichment pathways of keratinocytes and nerve cells lineages. For example, GO analysis showed that both keratinocytes and nerve cells were significantly enriched in neural crest cell development and positive regulation of epithelial to mesenchymal transition. This suggested that the migration and differentiation of neural crest cells might affect the migration of epithelial cells to mesenchymal cells, and also affect the differentiation of keratinocytes and nerve cells in the process of hornless to horn development. Moreover, hair follicle development and skin development were enriched in nerve cells lineage. Our analysis showed that the development of nerve cells may stimulate the differentiation of hair follicle cells, leading to changes in gene expression throughout development and playing an important role in the development of horn buds in dairy goats. As expected, GO analysis showed that keratinocytes and nerve cells both significantly enriched *KRT10,* and *APOE* in the development process, and with the development of a pseudo-timeline on the axis, these genes involved in the development were down-regulated in development later. *TOP2A*, *ACTA2*, *APOD*, and *SOD1* showed specific expressions in development different stages. *KRT10*, *APOE*, *TOP2A*, *ACTA2, APOD*, and *SOD1* may be the key candidate genes for the regulation of goat horn bud development.

To explore the dynamic changes of differential genes at developmental stages, we performed cell locus analysis on keratinocytes and nerve cell lines respectively. *KRT14* has been identified as a marker for keratinocytes both in humans and mice [45, 46, 58]. And *SOX2* has also been identified as a marker for nerve cells [59]. Interestingly, the expression of *KRT14* and *SOX2* was low in our study (see Additional file 5: Fig. S5c)*.* However, nerve cell differentiation was present at the most trajectory stages. It may be plausible that keratinocyte differentiation with the development of hair follicle was not synchronized. Previous studies have also demonstrated that the development of hair follicle gradually changes during fetal horn bud development [60]. To confirm the above hypothesis, immunofluorescence analysis of *LUM*, *SOX9*, *K15*, and *LEF1* was performed. As a transcription factor, *SOX9* not only plays a key role in chondrocyte differentiation and bone development but also regulates the proliferation and differentiation of epithelial stem cells [61]. Moreover, *K15* as a particular kind of keratin involves in the keratinization of epidermal cells as a cytoskeleton [62]. *LEF1* and *K15* in the outer layer of the epidermis were not homogeneous, with horned fetuses showing higher expression while hornless fetuses showing lower expression. *LEF1* and *LUM* were uniformly expressed in the interfollicular epidermis. At the same time, the expression patterns of *K15* and *LEF1* were more obvious and they were partially expressed in the epidermis (Fig. 4g; see Additional file 5: Fig. S5d, e). Unfortunately, we detected no changes in neural crest cell status in immunohistochemistry, indicating that neural crest cells presented a very brief process during horn bud development, and neural crest cells differentiated in E70 horned fetal dairy goat. Taken together, by dissecting horn-bud cell heterogeneity, our study emphasized that distinct signals orchestrate the asynchronous development of early horn buds. Furthermore, our trajectory inference analysis successfully recapitulated major cell fate decisions during dairy goat horn bud development, which enabled us to comprehensively study detailed developmental pathways involved in horn bud morphogenesis.

### The development process of horn buds showed distinct gene expression signatures

**Fig. 5:**
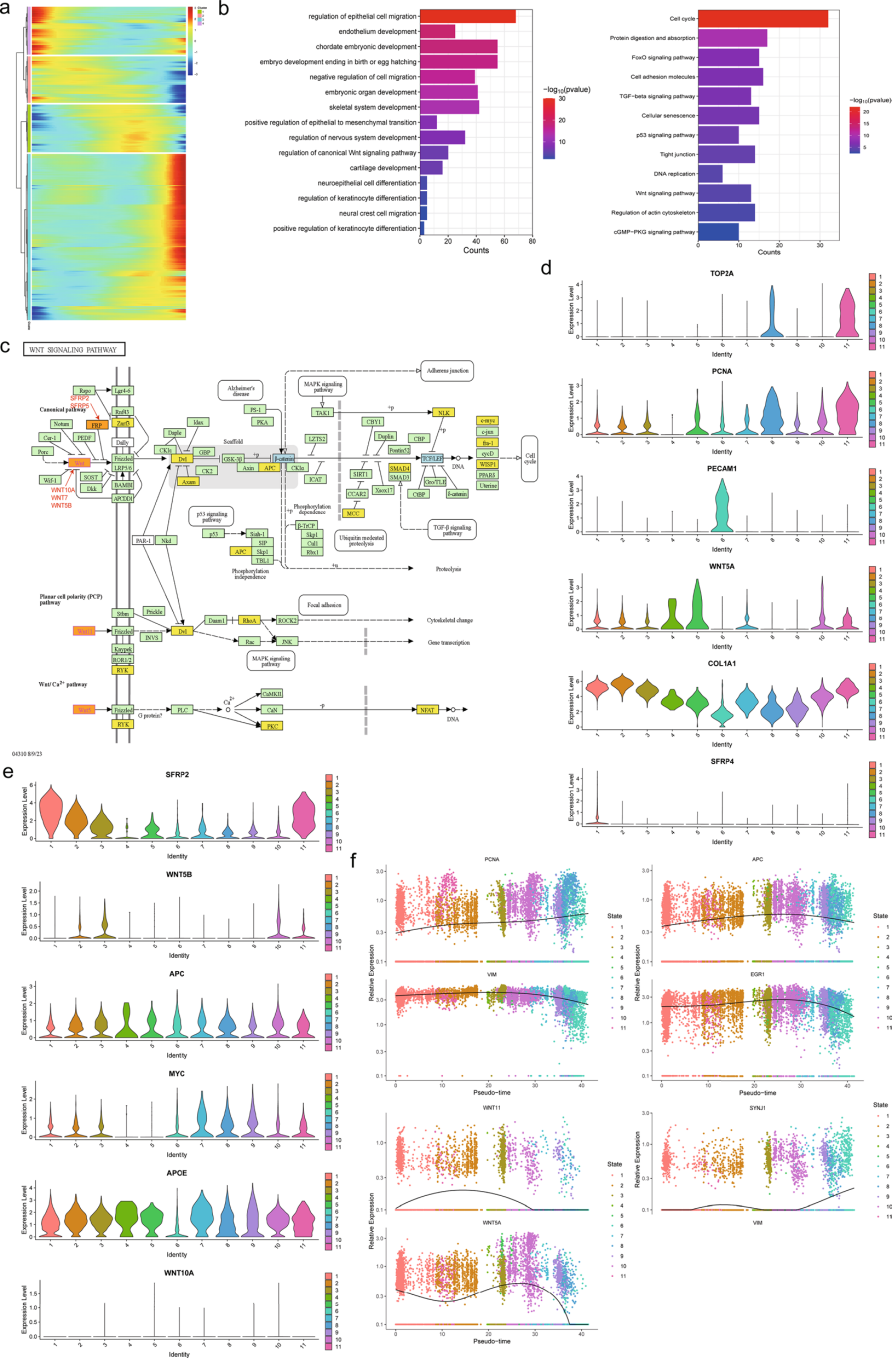
Summarizes the function of DEGs on horn bud development. **a** DEGs gene expression pattern heat map. **b** Enriched GO function and high expression gene visualization. **c** KEGG pathway map for Wnt signaling pathway. **d**, **e** Visualization of dairy goat orthologs of horn bud characteristic genes along pseudo-time in different gene-sets. **f** Expression trends of key candidate genes

After identifying changes in cell fate during hornless to horned development, we further focused on differential gene expression at each branch point, mainly involving heterogeneous changes in horn bud-associated cells. In short, changes in gene expression were major factors that led to different cell fates. In support of the draw order in cell trajectory, our analysis revealed the organization of genes that were differently expressed, which was closely related to the genes that were famous for participating in horn bud growth and skin development (Fig. 5a). Interestingly, the expression level of DEGs was a dynamic process, such as *CD47*, *HOXD8*, *LEF1*, and *SOX18* which were briefly up-regulated and then down-regulated were observed (see Additional file 6: Fig. S6a). To further investigate the function of the DEGs in the skin and horn buds, GO and KEGG analyses of those genes were performed.

In key branches, many gene functions, signaling pathways, and protein interactions were enriched for the formation mechanism of angle bud through GO biological function and KEGG signaling pathway analysis (Fig. 5b; see Additional file 6: Fig. S6b, c). To further investigate the function of the DEGs in the skin and horn buds, GO biological function and KEGG signaling pathway analysis of the top 10 markers of all clusters was carried out using the Metascape (see Additional file 9: Table S8). Finally, a total of 760 GO terms were significantly enriched. As expected, tissue morphogenesis, epithelial cell differentiation, skeletal system development, and skin development were significantly enriched GO terms (see Additional file 9: Table S9). The top 20 differentially expressed GO terms were shown in Fig. 5c. Interestingly, some DEGs were found to be mainly involved in the regulation of the cell cycle process such as “DNA replication preinitiation complex”, “chromosome organization”, and “positive regulation of transferase activity”. These terms proved that cell cycle, epithelial cell development, skeletal development, and neural crest cell development played an important role in the development of horn buds and promoted the transition from hornless to horned state in dairy goats horn bud development.

By KEGG analysis, some pathways related to horn bud growth and development were enriched, including cell cycle, cell adhesion molecules, regulation of actin cytoskeleton, PI3K-Akt signaling pathway, and WNT signaling pathway. *PECAM1*, *PCNA*, *COL1A1*, *TOP2A*, *SFRP4*, and *WNT5A* enriched these pathways (Fig. 5d). Results of KEGG analysis showed that the DEGs were mainly enriched in the WNT signaling pathway which included the *WNT11*, *WNT5B*, *APOE*, *SFRP2*, *APC*, and *MYC* genes (Fig. 5e). We performed the KEGG analysis by merging the genes from different gene sets of cell fate branches, the marker genes from this study, and the horn-related gene sets from previous studies. Interestingly, *WNT7B* and *WNT3* were also enriched in the Wnt signaling pathway, but the expression levels in our study were lower (see Additional file 6: Fig. S6d), suggesting that the biological functions of genes in the Wnt signaling pathway differed in different tissue development.

Subsequently, we integrated the gene sets and compared them with the DEGs identified in this study, including the horn-related genes and proteins of ruminant horns identified in previous studies [63, 64]. Our results screened key genes for a more detailed analysis, namely *RXFP2*, *FMOD*, *ZEB2*, *KRT10*, *SFRP2*, *SOX9*, *SFRP4*, and *SCUBE2* genes, which showed obvious differential expression changes during the horn bud formation (see Additional file 6: Fig. S6e). A large number of genes, signaling pathways, and proteins interactions were involved in the developmental mechanism of horn bud formation. Our study verified distinct gene expression signatures during the horned state to the horned state. Further identification of the roles of differentially expressed genes *WNT5A*, *SFRP1*, *WNT11*, *ERG1*, *VIM*, *APC*, and *PCNA* in key functions and pathways will be key to understanding the mechanism of horn bud development (Fig. 5f). In general, we provided an important reference for exploring the molecular genetic mechanisms of horn bud formation during early embryonic development in dairy goats.

### Elucidate the interactive characteristics among distinct cell types

Cell communication analysis showed that dermal cells had a higher number and strength of interactions with other cell types (see Additional file 7: Fig. S7). Among them, the number of cell interactions between dermal cells and epithelial cells, neural crest/chondrocytes and hair follicle/nerve cells, hair follicle cells, and horn bud associated/keratinocyte/stromal cells was higher (see Additional file 7: Fig. S7). However, the interaction strength between dermal cells and hair follicle/nerve cells, epithelial cells, neural crest chondrocytes, and hair follicle cells was significantly higher than that between dermal cells and cornea-related cells (see Additional file 7: Fig. S7). Cell communication analysis showed that multiple signaling pathways were found in ligand-receptor pairs during bud development, including WNT, Notch, TGFB and other pathways. In addition, further analysis of ligand-receptor pair interactions revealed that other cell types mainly act on dermal cells through ligand-receptor pairs. Dermal cells interact with other cell types for cell communication mainly through COL1A1-a1b1 and COL1A2-a1ba (see Additional file 8: Fig. S8). Dermal cells primarily exhibit strong interactions with cartilage cells and neural crest cells through the MDK-IGF1R signaling axis, while contrasting with NGFR-mediated pathways. Meanwhile, they interact strongly with hair follicle cells via the MDK-LRP1 pathway. We found that the ligand WNT5A primarily drives signals from dermal cells to epithelial cells, while horn bud/keratin-forming stromal cells transmit signals specifically to horn bud progenitors. Compared with other cells, dermal cells are the most important output cells of IGF2 signaling pathway. In addition, it was also observed that the *FN1* and *DLK1* genes were relatively highly expressed in the dermal cell subgroup, while non-canonical WNT signals such as WNT2 were highly expressed in horn bud associated/keratinocyte/stromal cells, suggesting that dermal cell cells may communicate with other cells through non-canonical WNT signaling. These data suggest that cell types with adipogenic potential, such as dermal cells, epithelial cells, and hair follicle cells may interact with each other through the COL1A1 and COL1A2 pathways. However, the detailed regulation and species specificity of these cell populations remain to be investigated in continuing horn bud development.

## Discussion

### Single-cell transcriptome reveals the regulation mechanism of different cell types in horn bud development

ScRNA-seq describes many complex biological processes in a detailed and comprehensive way since this approach offers strong analytical ability regarding cell heterogeneity, which helps to reveal functions of different cell types in complex tissues [65, 66]. As important domestic and model animals, the regulation of horn bud development during embryonic goat has not been investigated by scRNA-seq previously.

In this study, we systematically analyzed the regulation of gene expression profile of goat horn buds and constructed a single-cell atlas to reflect dairy goat horn bud development. ScRNA-seq analysis revealed new insights into the cellular heterogeneity and major cell fate decisions taking place during in-utero dairy goat horn bud morphogenesis. We identified the evolutionarily conserved and species-specific regulators in goats based on enrichment in the expression of marker genes previously reported in mice, humans, or other mammals [67, 68]. Previous studies have found that *LUM*, *LEF1*, and *COL1A1* are important transcriptional factors and are highly expressed in skin of mice, cashmere goat, and humans [20, 69, 70]. Our study found that these genes were also expressed in the dermal cells and hair follicle cells of goat fetuses, suggesting that these factors were most likely evolutionarily conserved. *COL1A1* and *COL1A2* play critical roles in mammalian skin cell interactions [70–72]. Similarly, these two proteins serve as key ligands in the cross-talk between major cell types during goat horn bud development. Other than these factors, we also found transcriptional factors (*TOP2A*, *SOX2*, *SOX18*, and *KDR*) that were highly expressed in the key cell clusters (epithelial cells, nerve cells, and endothelial cells) of goat fetuses [59, 73, 74], suggesting these factors were probably also involved in the regulation of horn bud development.

As far as we know, this is the first study to comprehensively delineate the molecular profiles of various cell types and reveal major cell fate decisions during horn bud development in goat fetuses. Our study also provided evidences that differentiation signals of different cells related horn formation may participate in orchestrating the development of horn buds in dairy goats. More importantly, by analyzing cluster-specific gene expression profiles, the current data provide a valuable resource for future studies in dairy goat horn buds and an in-depth understanding of horn development.

### Changes of key cell differentiation and gene expression promote the formation and development of horn buds

The embryonic period is crucial for the differentiation and formation of the horn structure. According to previous work, the appearance of the horn primordium and the horn formation takes place at 51 days of embryonic development. The angular protrusion was obvious until 60 days, the horn angle thickened at 70 days and the keratinization began at 80 days [75, 76]. We hypothesized that there would be great differences in the formation and development of the skin tissue in the horn bud area of horned and non-horned fetuses. In this study, the histological results showed that the horn buds were significantly different from the forehead skin, showing a thicker epidermis and more layers of epithelial cells, but little difference was displayed in E50. This was in agreement with previous studies [60, 77]. At the same time, vacuolated keratinocytes previously found in bovine fetal horn buds were also observed in our study [75]. However, in a previous study of merino sheep, the vacuolated keratinocytes were not found in the development of horn buds from E75 embryos to adulthood. We compared the histomorphology of E50, E60, and E70 goat fetal horn bud skin tissue. The results indicated that E60 has begun to show multiple layers of vacuolated keratinocytes during the process of horn bud formation in goats. Moreover, Davis found that at the initial stage of horn bud formation, hair follicles are not present in the horn bud, but keep growing and developing along with the horn bud, and the development of the hair follicle would gradually mature [52, 78]. Our study found that the process of horn formation was accompanied by the development and differentiation of hair follicles. In addition, we also found that the differentiation of keratinocytes in E50-E70 fetuses at different developmental stages showed a dynamic change process, while hair follicles were more obvious in hornless fetal goats. The skin tissue structure of the horn bud in E70 was also a nerve bundle, but there was no obvious structure before this. Therefore, the development of horn buds in fetuses was accompanied by the differentiation of nerve cells and hair follicles during embryonic development.

Similarly, in the early characterization, we found that the skin cell types of the early embryos (E50) of dairy goats resembled each other, but the specificity of the different stages became more apparent as development progressed. In this study, we further analyzed the changes of key cell clusters and supposed the sequence of cell clusters according to the pseudo-time trajectory. We found that branch 1, as a key point, played an important role in horn bud development. Nerve cells acted as a starting signal to stimulate hair follicle cells and changed hair follicle cell gene expression. These changes stimulated dermal cells to continuous and specific differentiation, causing epidermal cells to reshape, and jointly promoting the development of goat embryonic horn through the interaction of key cells in various branches. Previous studies have proved that the development of horns is closely related to the differentiation of dermal and epidermal tissues [52, 79], and the formation of horns is also accompanied by the differentiation of hair follicle cells and nerve cells [52]. Neural crest cells play an important role in horn development, promoting epithelial to mesenchymal cell differentiation [2, 9, 10]. Our study also demonstrated that neural crest cells indicated a key regulatory role in cell fate changes. Moreover, we also found that keratinocytes might play an important role in the development of horn bud. Although our data here delineated the asynchronous development of E70 dairy goat fetuses regarding horn-bud development, the detailed molecular mechanisms underlying were not investigated. Future studies might focus on such topics, which could provide us with new insights into horn bud biology and regeneration.

### Revealing the expression of key genes in horn bud development

ScRNA-seq was performed in this study to investigate the gene expression profile of dairy goat horn buds during the embryo period. We found some major DEGs in the horn bud and forehead skin tissue, suggesting that horn development may be regulated by many key genes during embryonic development. In light of previous studies of genes involved in horn formation in sheep, goats, and cattle, we validated the genes involved and screened for candidate genes associated with goat horns. These candidate genes may play a role in horn development, including epidermal keratinocyte differentiation, epithelial–mesenchymal transition, migration, and differentiation of neural crest cells. Therefore, their functions need to be further investigated.

Structural variants at about 129 Mb on chromosome 1 in goats are now presumed to affect the transcription of at least four genes. Here, *FOXL2* is a female sex-determining gene in the goat [80]. This gene involves in horn development and plays negative regulation role of horn bud differentiation in goats [2]. The *ERG* gene is involved in bone development, embryonic development, cell proliferation and differentiation, angiogenesis, and hematopoietic processes, so normal horn growth of goats may also be affected by the *ERG* gene [81, 82]. The *KCNJ15* gene is highly expressed in mouse follicle-associated epithelium cells [83], and also exists in normal mouse liver [84], suggesting that the *KCNJ15* gene may be involved in the development of goat ovarian function and indirectly affect the formation of goat horns. *PISRT1* expression decreased while *SOX9* expression increases in the gonads of intersexual goats and its gene polymorphism is closely associated with intersexual goats [8, 14]. However, we did not find any possible regulation of the *FOXL2*, *PISRT1*, *ERG*, *and KCNJ15* genes associated to horn formation in our study. Therefore, we will further examine other factors that regulate these genes in more detail to clarify this.

*SOX9* can also be identified as a marker gene of cranial neural crest chondrogenesis lineage [8]. The interaction of the *SOX9* gene and the *FOXL2* gene regulates the formation and development of horns, further revealing the genetic pathway of horn budding differentiation [33, 85]. Interestingly, *FOXL2*, is involved in horn bud differentiation [33] and associated with horn growth in goats [86]. However, a non-specific expression of *FOXL2* pattern was shown in our study. While the specific expression of *SOX9* was demonstrated in our study, which further verified that *SOX9* could regulate the development of neural crest cells and thus play a positive role in the regulation of horn bud development. In addition, *TGF-β*, *SNAI2*, and *NGFR* are associated with neural crest cell migration and differentiation pathways and may play a key role in the evolution and development of horns. In our study, we verified the expression changes of these genes during the development of goat horns, thus proving that they might promote the development of horn buds. In addition, our results demonstrated that *ZEB2* and *SCUBE2* are related to epithelial cell to mesenchymal cell transformation and are involved in hair follicle and horn bud differentiation [87]. The *RXFP2* gene plays an important role in controlling horn bud size, and the confirmation of *RXFP2* as the main candidate gene for controlling horn bud development indicates that *RXFP2* plays a key role in the regulation of horn development [88]. In this study, *RXFP2* was also differential expression in different state of development of goat horn bud. Genes in the HOXD family have also been shown to play a role upstream of the horn gene regulatory network and are key genes in the initiation of horn development [64]. In our study, we found that *HOXD8* was highly expressed during horn development. Moreover, we found new genes (*EGR1*, *BMP4*, *CUX1*, and *APOD*) that might play key roles in the regulation of horn bud development.

According to our KEGG analysis, the identified genes were mainly enriched in the Wnt signaling pathway, which has also been shown to play vital roles in epidermal development, stem cell self-renewal, and wound healing [91–94]. At the same time, the Wnt signaling pathway is essential for regulating the fate, migration [22, 95], and proliferation of cranial neural crest cells [95–98]. Overall, these findings would suggest that the Wnt signaling pathway was important in the development and formation of horns.

## Conclusions

In the present study, we have investigated the cell fate trajectory and key gene expression profile of horn buds in goat fetuses using scRNA-seq: 1. By comparing histological features of hornless and horned fetuses at E70, scRNA-seq revealed the cellular heterogeneity of epithelial cells, dermal cells, nerve cells, and hair follicle cells, as well as the trend of fate changes of these cells in different states. 2. Integration of pseudotime inference and immunohistochemistry demonstrated that Branch 1 represents the critical signaling cascade initiating horn bud formation. This finding highlights the pivotal roles of dermal-epithelial crosstalk and specific transcriptional programs in driving the transition from hornless to horned states. 3. We characterized state transitions in keratinocytes and neural cells, uncovering their active roles in regulating horn bud outgrowth. Temporal immunohistochemical analysis confirmed asynchronous developmental patterns among these cell populations. 4. GO and KEGG enrichment analyses identified neural crest development and keratinocyte differentiation as key biological processes. Differential gene expression networks were further interrogated to prioritize candidate markers (e.g., *EGR1*, *ZEB2*, *SFRP2*, *KRT10*, *FMOD*, *CENPW*, *LDB1*, and *TWIST1*) influencing horn bud morphogenesis.

## Supplementary Information

Additional file 1: Figure S1. Expression of different cell clusters and marker genes in the formation of horn buds. (a) Apparent comparison of E70 fetal goat. (b) PCA identified the top 20 PCs at *P*<0.05. (c) Relationship between the number of molecules and captured genes. (d) Number of cells per cell cluster after quality control. (e) Overview of major cell clusters marker gene expression. (f) The expression of maker gene was based on state.

Additional file 2: Figure S2. RNA Velocity Visualization on Monocle Trajectories. (a) RNA velocity vectors projected onto the trajectory of horn bud cell lineage. (b) RNA velocity vectors projected onto the trajectory of forehead skin cell lineage.

Additional file 3: Figure S3. Pseudo-time trajectory analysis cell state transition. (a) All cell state transition and branch analysis. (b)Tree trajectory based on cluster. (c) Cell density based on cell type. (d) Branches trajectory based on state.

Additional file 4: Figure S4. Pseudo-time trajectory analysis delineated molecular profiles during branch1. (a) Overlap between gene lists. (b) Biology processes of Top20 GO enrichment. (c) GO biological processes or KEGG pathway description. (d) Genetic interaction between gene list. (e) Changes in expression of *LEF1* and *LUM* genes. (f) Immunofluorescence analysis of *LEF1* and *LUM* expression in the skin and horn bud.

Additional file 5: Figure S5. Differential gene expression and functional enrichment in keratinocytes and nerve cells. (a) Gene function enrichment in keratinocytes. (b) Gene function enrichment in nerve cells. (c) Trend of gene expression in keratinocytes and nerve cells. (d) Immunofluorescence analysis of *SOX9* and *K15* expression in the skin and horn bud. (e) Immunofluorescence analysis of *LEF1/K15* and *LUM/K15* expression in the hornless fetal skin.

Additional file 6: Figure S6. Differential gene expression and functional enrichment in horn bud development. (a) Trends of differential gene expression. (b) Function enrichment of differential gene expression. (c) Changes in expression of *LEF1* and *LUM* genes. (d) Expression of candidate genes based on states.

Additional file 7: Figure S7. Interaction boxplot among major cell types in horn bud tissue

Additional file 8: Figure S8. Ligand-receptor pathways mediating interactions among different cell types in goat horn buds.

Additional file 9: Table S1. Different expression genes (DEGs) in dairy goat embryos. Table S2. Single cell data set quality analysis. Table S3. All markers of cell number of clusters. Table S4. Significant genes in branch 1.Table S5. GO for different clusters of nerve cells and performed KEGG function analysis of epithelial and dermal cell fate. Table S6. Summary of GO biological function and KEGG signaling pathway analysis of keratinocyte. Table S7. Summary of GO biological function and KEGG signaling pathway analysis of nerve cells. Table S8. Significant genes in the top 10 of all clusters. Table S9. Summary of GO biological function and KEGG signaling pathway analysis of key branches.

## Data Availability

The dairy goat scRNA-seq data used in this study have been deposited in the Genome Sequence Archive in National Genomics Data Center, China National Center for Bioinformation/Beijing Institute of Genomics, Chinese Academy of Sciences (GSA: CRA012984) that are publicly accessible at https://ngdc.cncb.ac.cn/gsa. All original code has been deposited at Mendeley and is publicly available as of the date of publication. DOIs are listed in the key resources table.
