# Supplementary figures and images for: Multiple cell types guided by neurocytes orchestrate horn bud initiation in dairy goats
Source: Genet Sel Evol. 2025 Jul 1;57:34. doi: 10.1186/s12711-025-00981-3 (PMC12220536; doi:10.1186/s12711-025-00981-3)

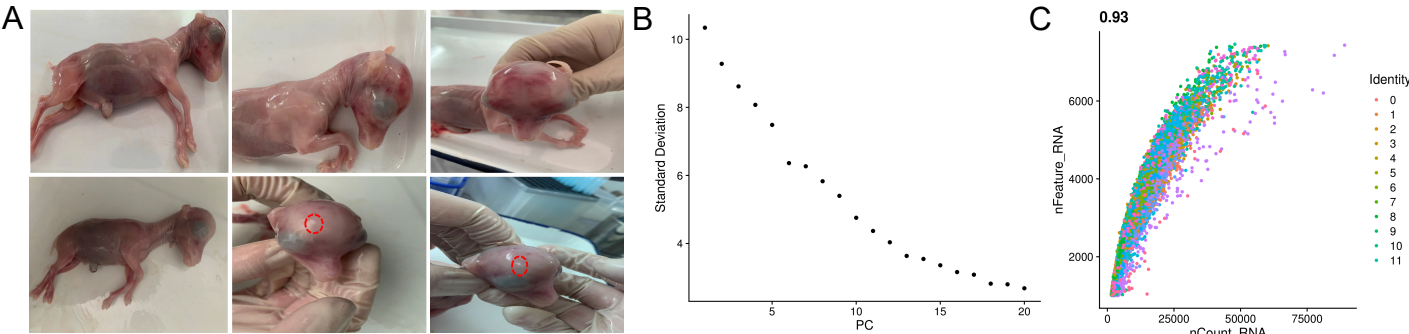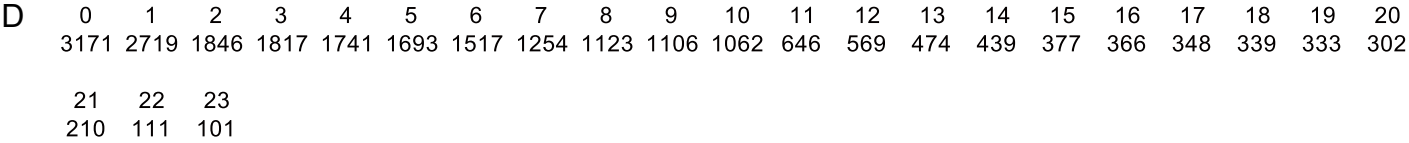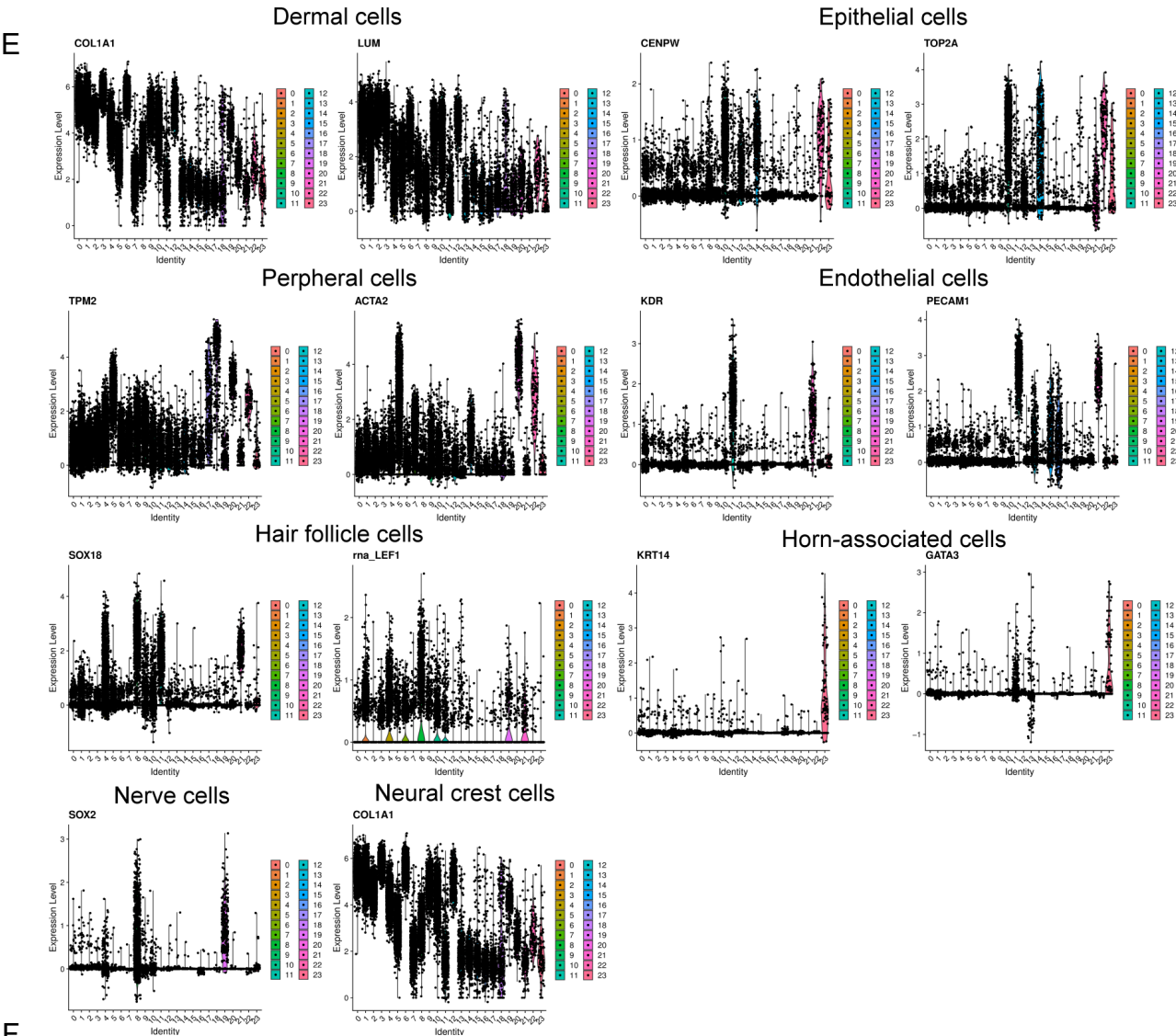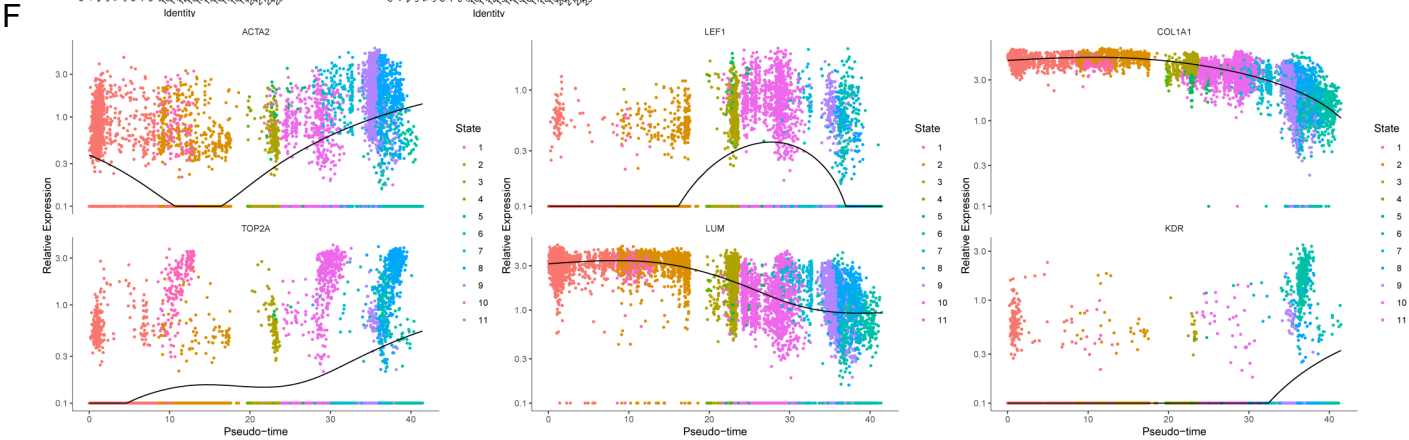

Supplement: Supplementary file 1 — Additional file 1: Figure S1. Expression of different cell clusters and marker genes in the formation of horn buds. (a) Apparent comparison of E70 fetal goat. (b) PCA identified the top 20 PCs at P<0.05. (c) Relationship between the number of molecules and captured genes. (d) Number of cells per cell cluster after quality control. (e) Overview of major cell clusters marker gene expression. (f) The expression of maker gene was based on state. [file 12711_2025_981_MOESM1_ESM.pdf]

**a****Cell Velocity**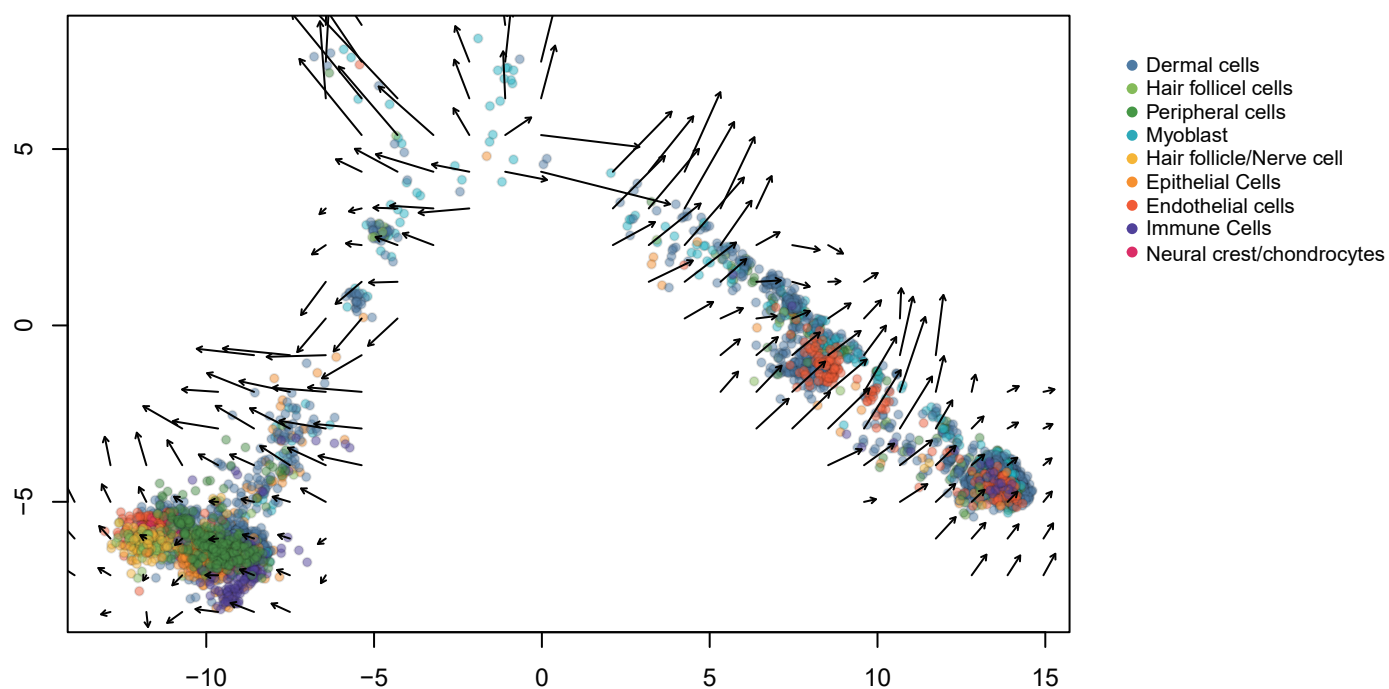**b****Cell Velocity**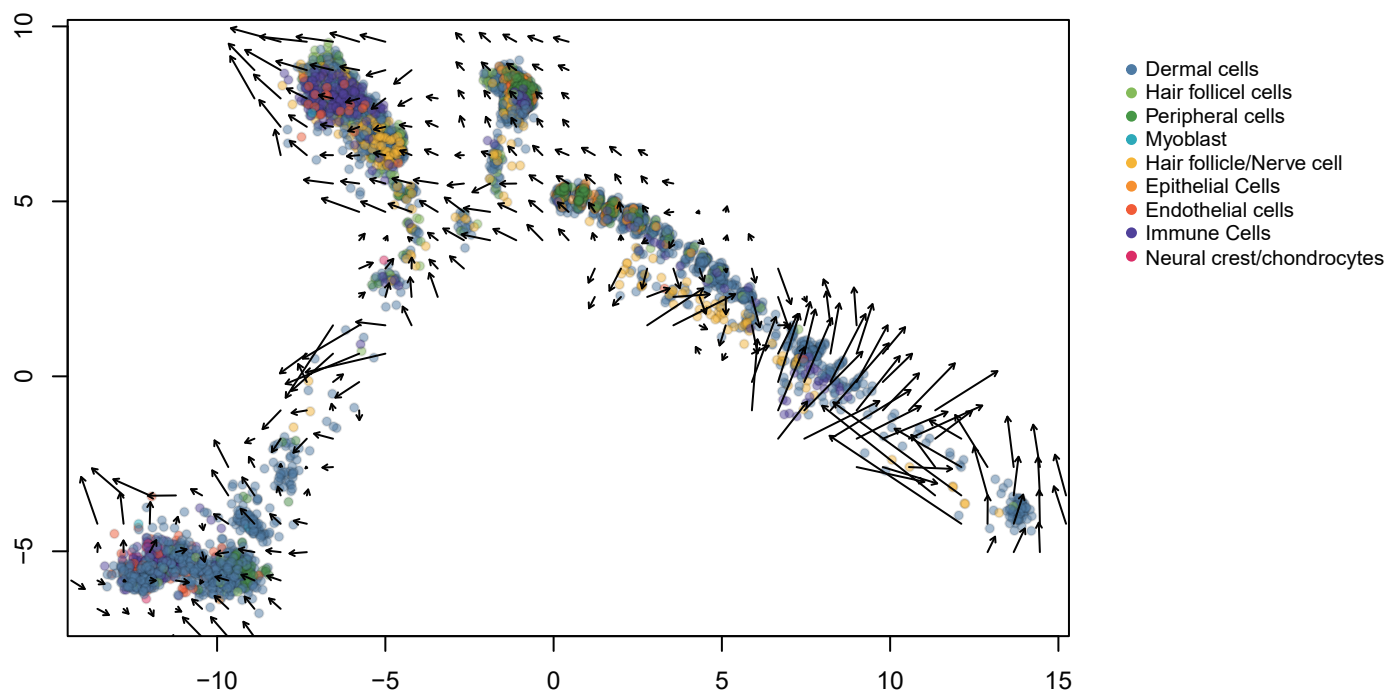

Supplement: Supplementary file 2 — Additional file 2: Figure S2. RNA Velocity Visualization on Monocle Trajectories. (a) RNA velocity vectors projected onto the trajectory of horn bud cell lineage. (b) RNA velocity vectors projected onto the trajectory of forehead skin cell lineage. [file 12711_2025_981_MOESM2_ESM.pdf]

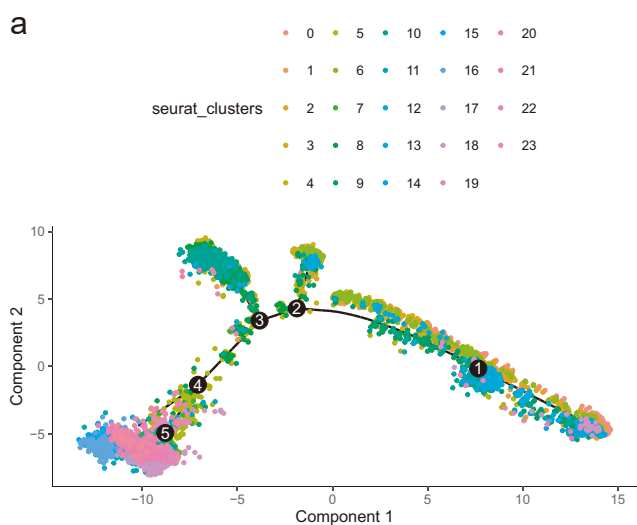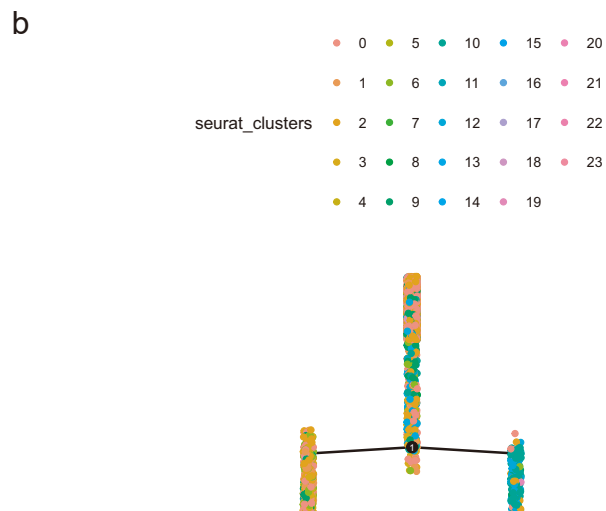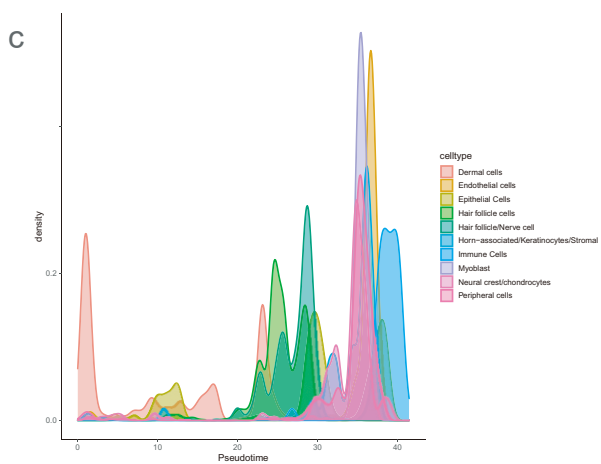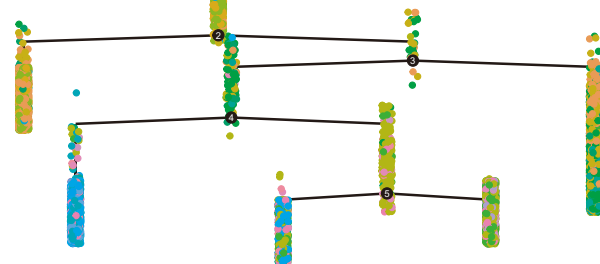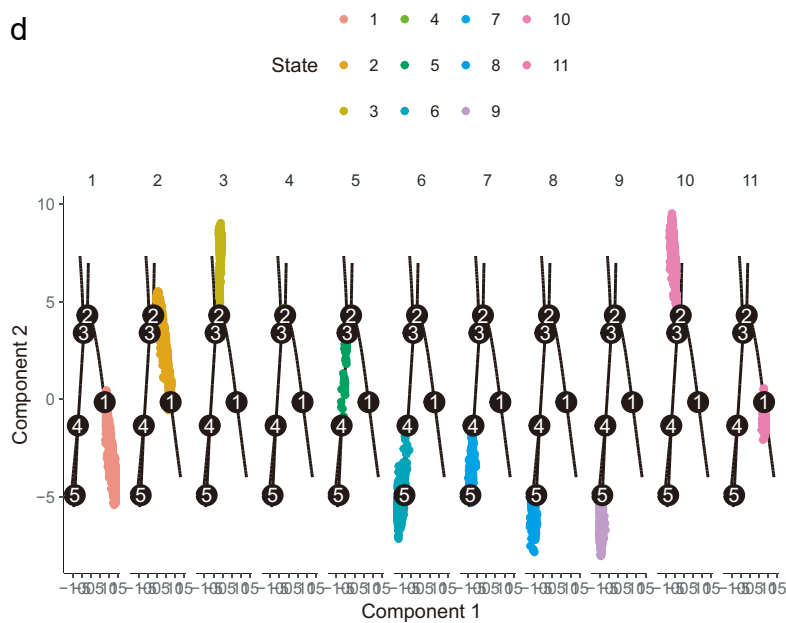

Supplement: Supplementary file 3 — Additional file 3: Figure S3. Pseudo-time trajectory analysis cell state transition. (a) All cell state transition and branch analysis. (b)Tree trajectory based on cluster. (c) Cell density based on cell type. (d) Branches trajectory based on state. [file 12711_2025_981_MOESM3_ESM.pdf]

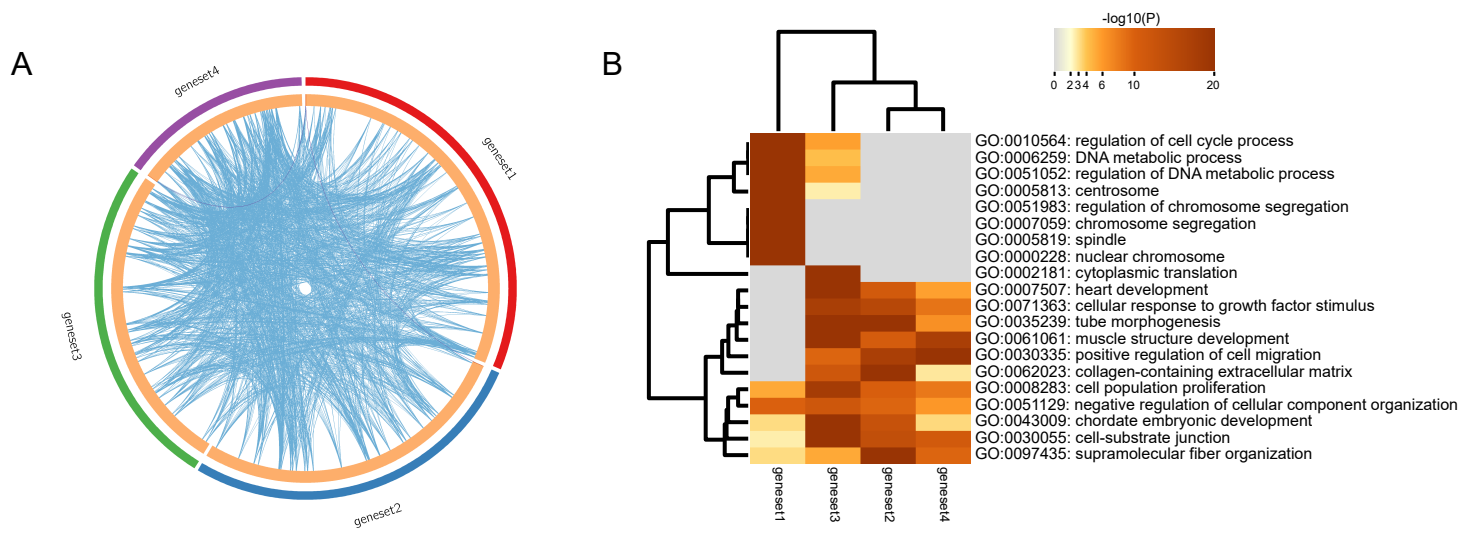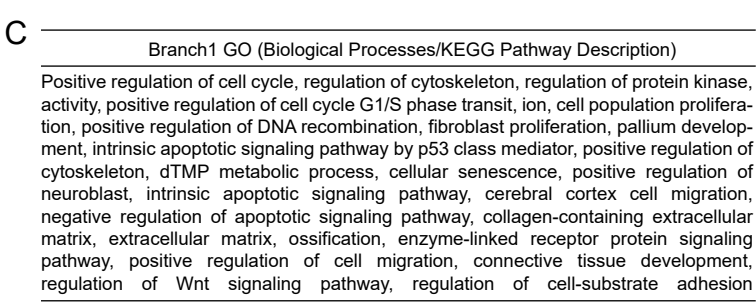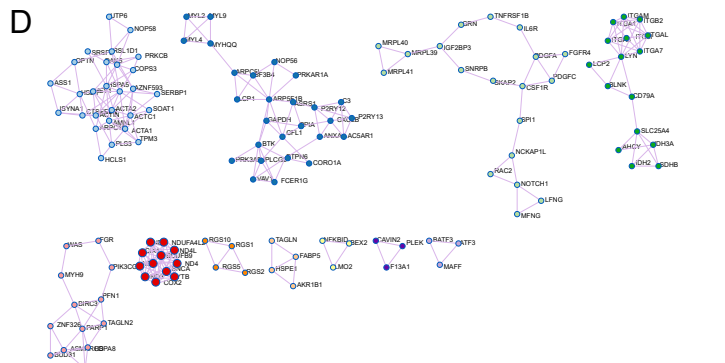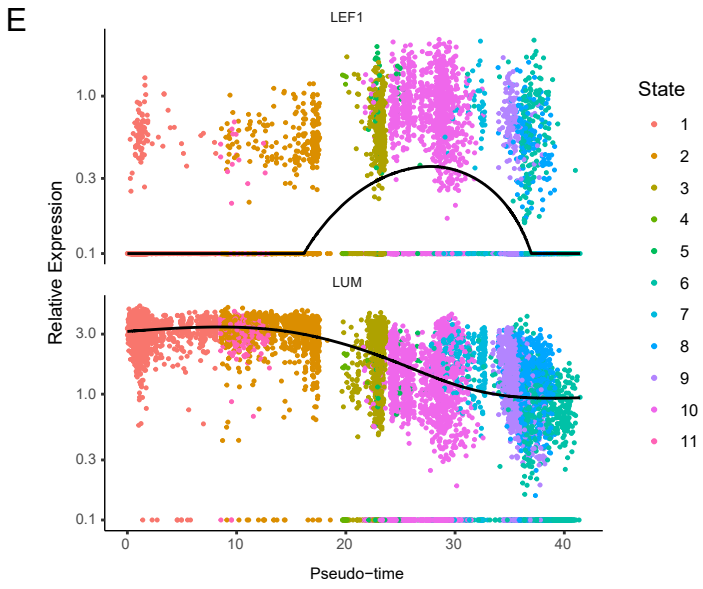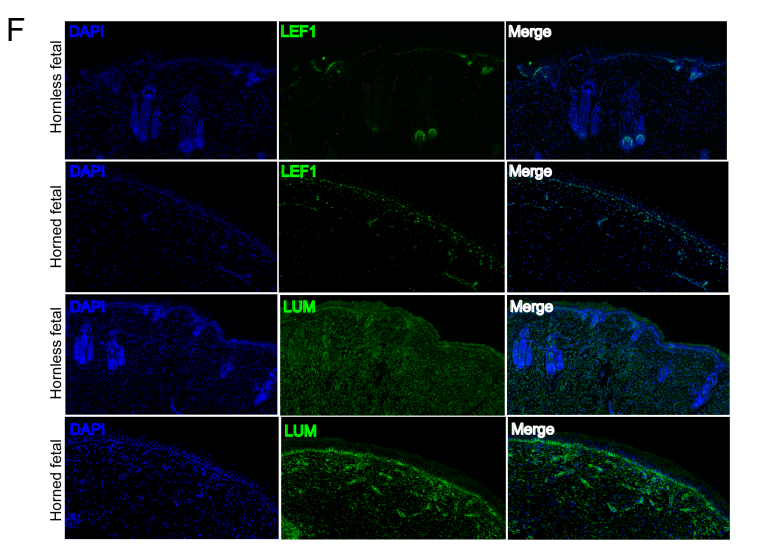

Supplement: Supplementary file 4 — Additional file 4: Figure S4. Pseudo-time trajectory analysis delineated molecular profiles during branch1. (a) Overlap between gene lists. (b) Biology processes of Top20 GO enrichment. (c) GO biological processes or KEGG pathway description. (d) Genetic interaction between gene list. (e) Changes in expression of LEF1 and LUM genes. (f) Immunofluorescence analysis of LEF1 and LUM expression in the skin and horn bud. [file 12711_2025_981_MOESM4_ESM.pdf]

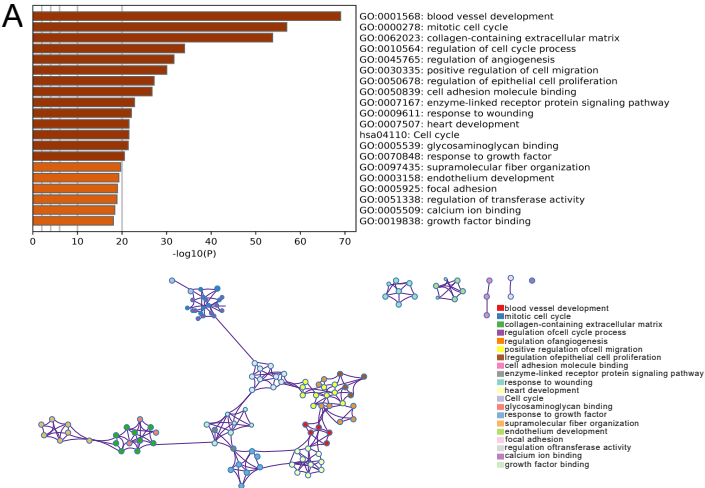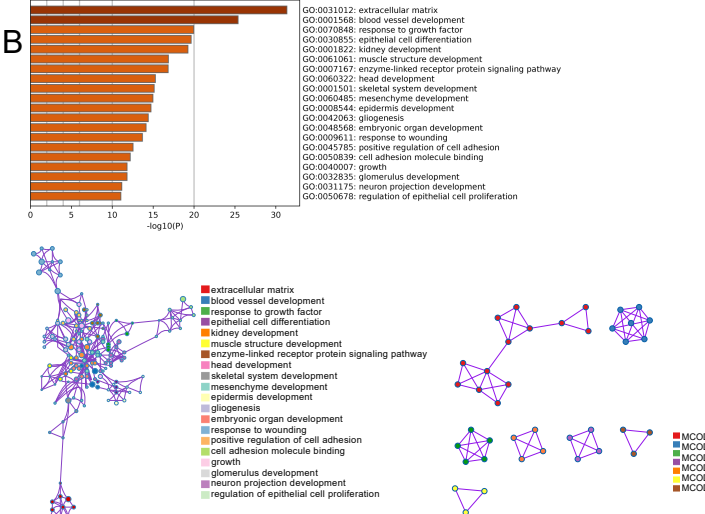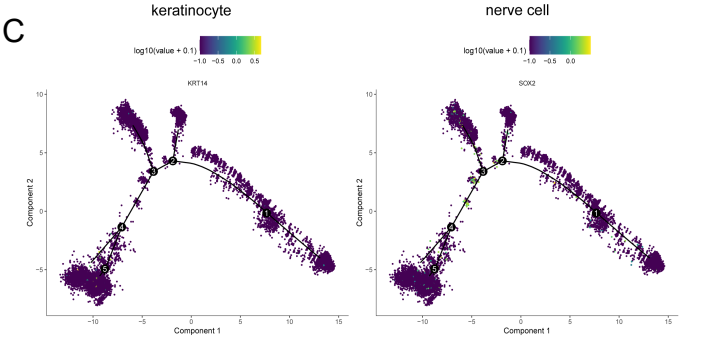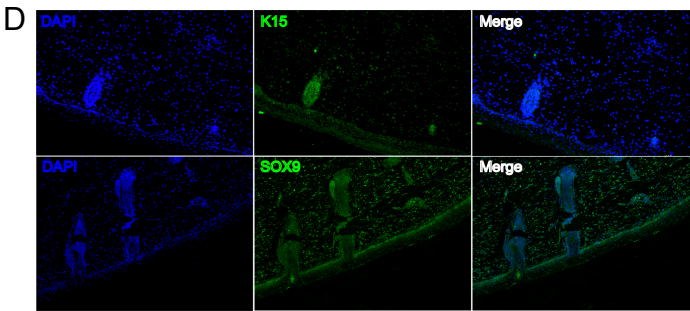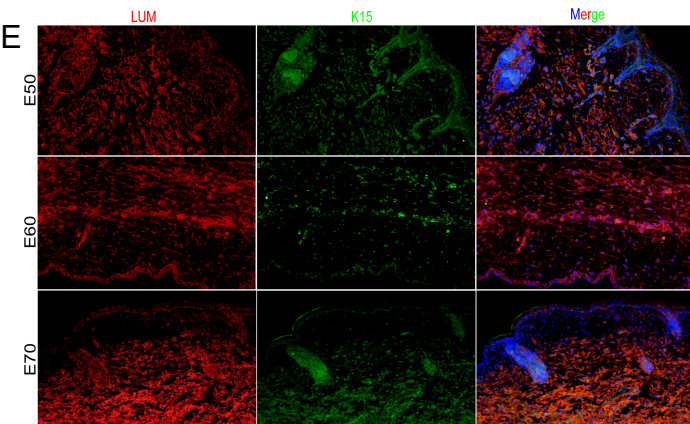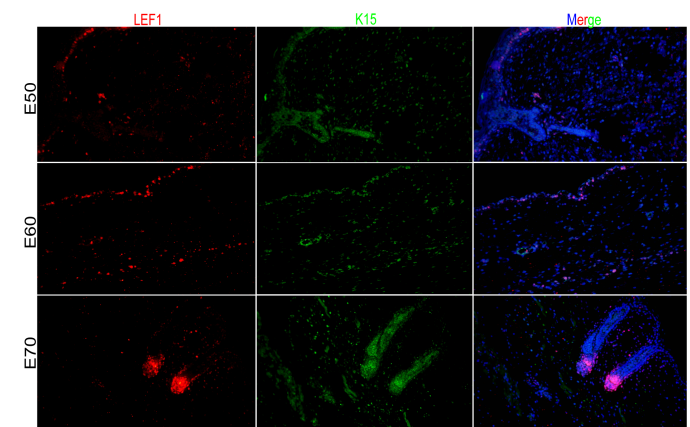

Supplement: Supplementary file 5 — Additional file 5: Figure S5. Differential gene expression and functional enrichment in keratinocytes and nerve cells. (a) Gene function enrichment in keratinocytes. (b) Gene function enrichment in nerve cells. (c) Trend of gene expression in keratinocytes and nerve cells. (d) Immunofluorescence analysis of SOX9 and K15 expression in the skin and horn bud. (e) Immunofluorescence analysis of LEF1/K15 and LUM/K15 expression in the hornless fetal skin. [file 12711_2025_981_MOESM5_ESM.pdf]

A

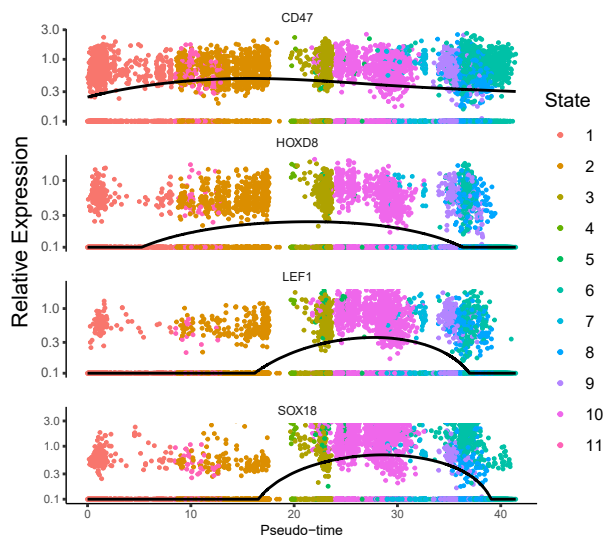

B

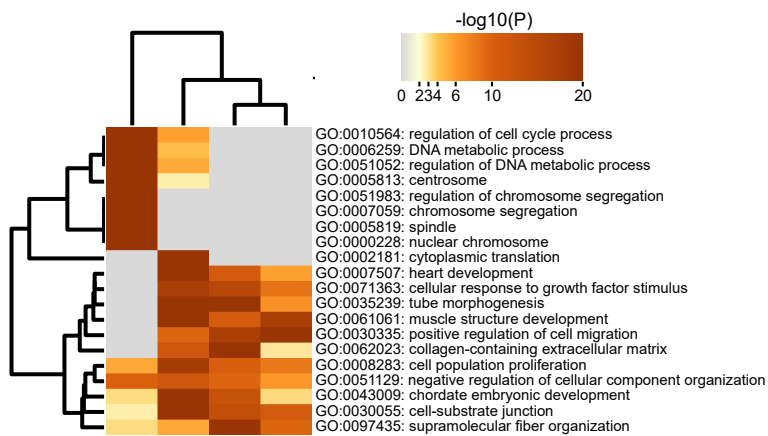

C

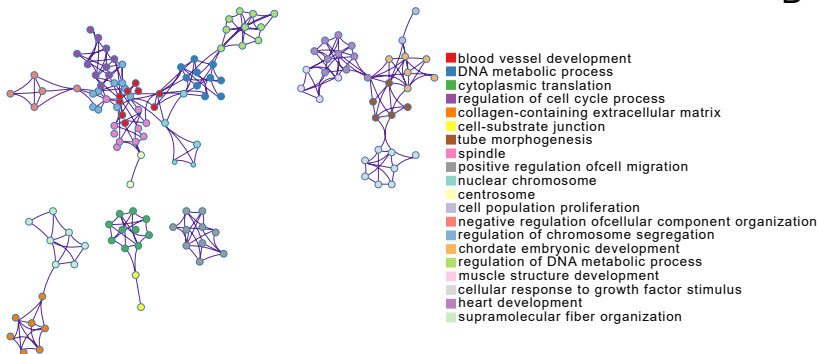

D

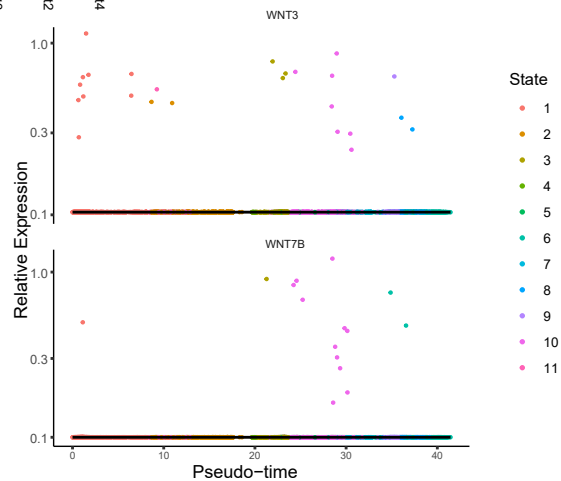

E

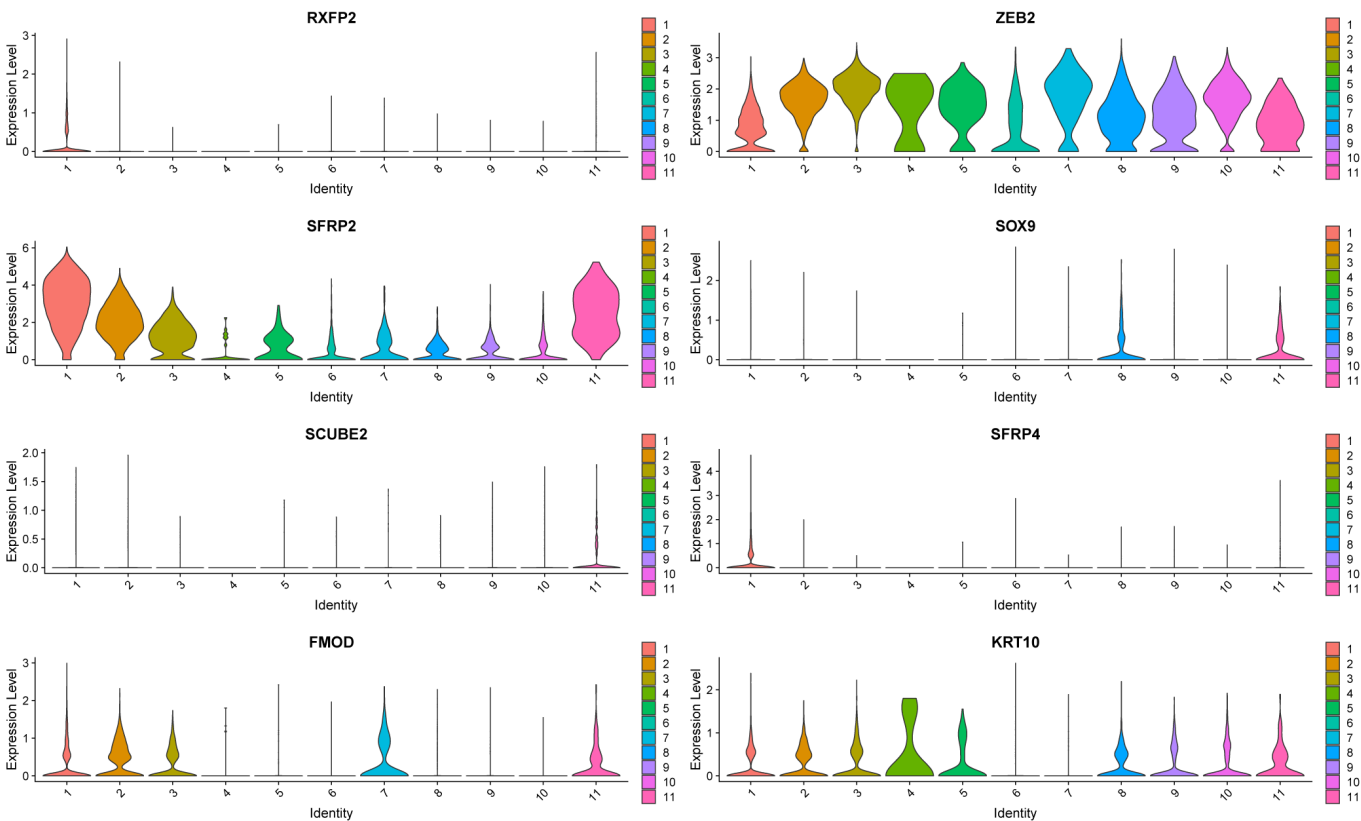

Supplement: Supplementary file 6 — Additional file 6: Figure S6. Differential gene expression and functional enrichment in horn bud development. (a) Trends of differential gene expression. (b) Function enrichment of differential gene expression. (c) Changes in expression of LEF1 and LUM genes. (d) Expression of candidate genes based on states. [file 12711_2025_981_MOESM6_ESM.pdf]

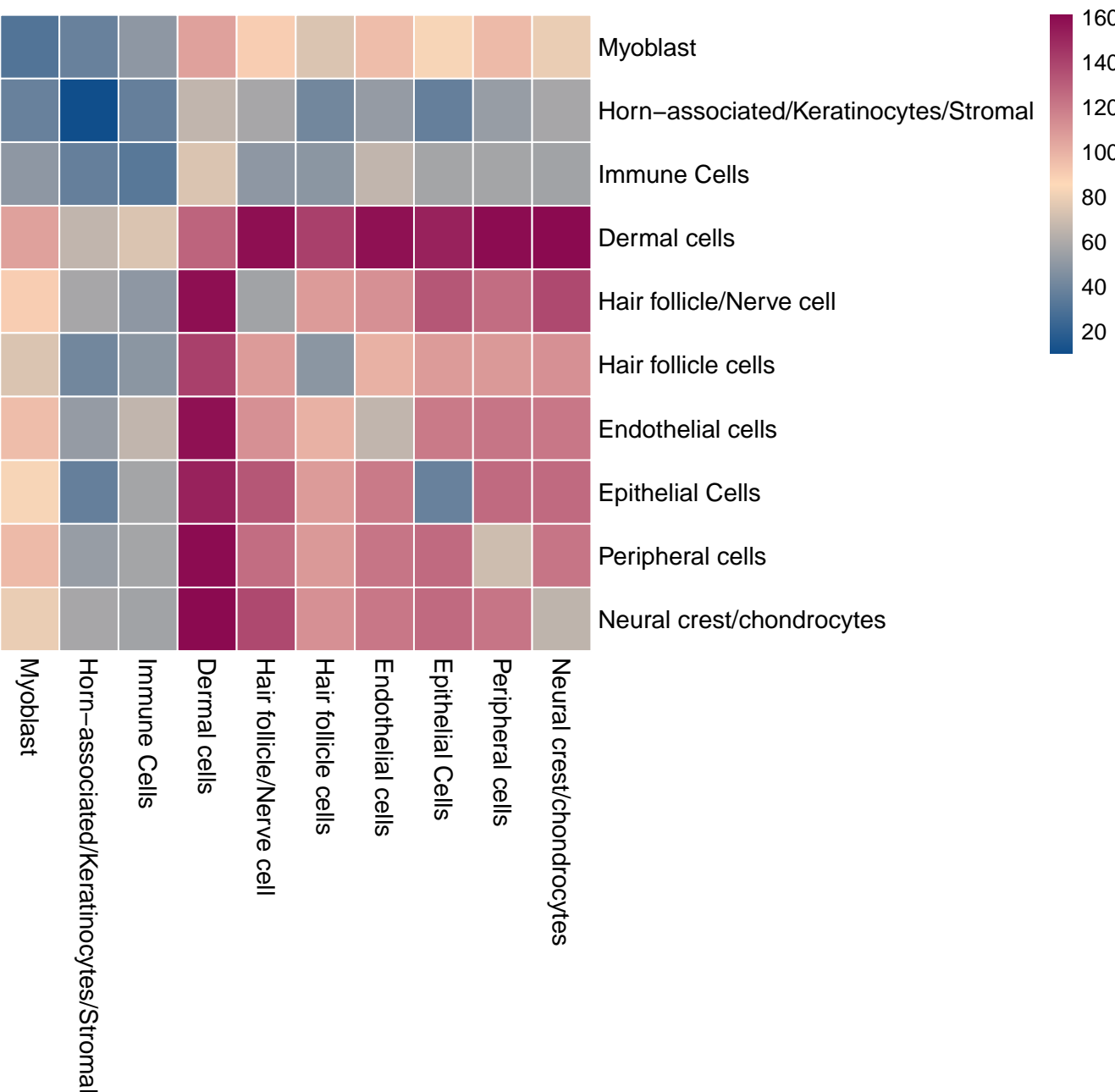

Supplement: Supplementary file 7 — Additional file 7: Figure S7. Interaction boxplot among major cell types in horn bud tissue [file 12711_2025_981_MOESM7_ESM.pdf]
