# Supplementary material for: Multiple cell types guided by neurocytes orchestrate horn bud initiation in dairy goats
Source: Genet Sel Evol. 2025 Jul 1;57:34. doi: 10.1186/s12711-025-00981-3 (PMC12220536; doi:10.1186/s12711-025-00981-3)

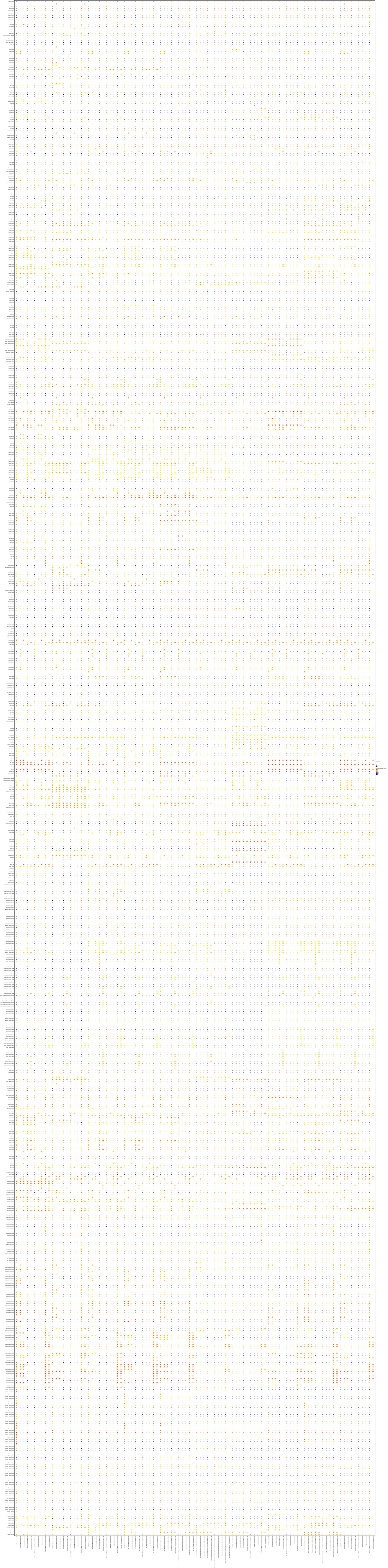

Supplement: Supplementary file 8 — Additional file 8: Figure S8. Ligand-receptor pathways mediating interactions among different cell types in goat horn buds. [file 12711_2025_981_MOESM8_ESM.pdf]
